# From Lipid Regulation to Neuroprotection: Multitarget (Benzo)thiazine Derivatives as Promising Leads

**DOI:** 10.3390/molecules30234542

**Published:** 2025-11-25

**Authors:** Ariadni Tzara, Andrea Andreou, Angeliki P. Kourounakis

**Affiliations:** Department of Medicinal Chemistry, School of Pharmacy, National and Kapodistrian University of Athens, 15771 Athens, Greece

**Keywords:** multitarget, antioxidant, anti-inflammatory, anti-hyperlipidemic, neuroinflammation, neurodegeneration, cardiovascular

## Abstract

Neurodegenerative and cardiovascular disorders share multifactorial origins, including oxidative stress, (neuro)inflammation, and lipid dysregulation—factors often addressed independently by single-target therapies. In this study, we report a rational multitarget approach through the design and synthesis of novel (benzo)thiazine derivatives that integrate antioxidant, anti-inflammatory, and antihyperlipidemic functionalities within a single molecular framework. The compounds were obtained in good yields via 3–7 step synthetic routes and evaluated through complementary in vitro and in vivo assays. Several derivatives displayed potent inhibition of lipoxygenase (IC_50_ < 100 μM), significant reduction in carrageenan-induced edema (up to 60%), strong free radical scavenging and lipid peroxidation inhibition, as well as effective iron chelation. In vivo, most derivatives enhanced total antioxidant capacity (by 50–800%) and significantly improved plasma lipid profiles in mouse, while almost all compounds increased the plasma antiatherogenic index by more than 100% with selected compounds exceeding 600%. Notably, several molecules also showed moderate acetylcholinesterase inhibition, suggesting preliminary neuroprotective potential. Altogether, these multifunctional (benzo)thiazine derivatives represent promising lead structures for the development of agents targeting the complex interplay of oxidative, inflammatory, and metabolic pathways underlying neurodegenerative and cardiovascular diseases.

## 1. Introduction

Neurodegeneration is a complex, multifactorial disease, characterized by significant neuronal cell dysfunction. This condition can evolve slowly or rapidly, resulting in neuronal loss and therefore cognitive decline. The most common chronic neurodegenerative diseases are Alzheimer’s and Parkinson’s disease, amyotrophic lateral sclerosis (ALS), multiple sclerosis (MS), and Huntington’s disease [1]. Major risk factors for neurodegenerative disorders are a combination of genetic and environmental parameters, such as altered protein morphology, oxidative stress, DNA damage, neurotrophin dysfunction, neuroinflammation, age, mitochondrial dysfunction, metabolic cardiovascular diseases, metal ion homeostasis, etc. [2,3,4]. Among these parameters, oxidative stress, inflammation, and hyperlipidemia have a significant and detrimental contribution to the establishment and progression of neurodegeneration.

The theory of Reactive Oxygen Species (ROS)-related aging and neurodegeneration is based on the brain tissue’s susceptibility to oxidative stress. The vast consumption of oxygen by the brain for its metabolic needs leads to formation of oxygen and hydroxyl reactive species. At the same time, brain tissue has large amounts of free fatty acids at the synapses lipid membranes, which are easily peroxidized. These facts, as well as the abundance of metal ions in the brain, are parameters that favor the establishment of oxidative conditions in this tissue, affecting neurons irreversibly [5,6,7]. The main source of neuronal oxidative stress are mitochondria, due to the respiratory chain, which produce hydroxyl radicals and anions via the Fenton reaction. Under normal circumstances, ROS are deactivated by the endogenous antioxidant system. However, under conditions of oxidative stress, ROS attack biological macromolecules, such as phospholipid membranes, proteins, and nucleic acids, altering their function and inducing the mitochondrial secretion of pro-apoptotic proteins [6,8].

Dysregulation of metal ion homeostasis is a common factor in many disorders, including neurodegeneration. Many studies have confirmed that an excess of metal ions, such as iron, copper, zinc, and mercury, poses a significant risk for the establishment and progression of Alzheimer’s disease [9,10,11]. In particular, regarding iron ions, under normal circumstances they participate in various cellular functions, such as enzymatic catalysis, oxygen transfer, development and differentiation of cells, and mitochondrial respiration. However, iron overload in the brain contributes to oxidative stress and protein degradation [9]. Studies have confirmed that neurodegenerative diseases are correlated with increased levels of iron at the damaged brain area, as a result of various mechanisms [12].

Further, neuroinflammation plays a pivotal role in neurodegeneration. The chronic inflammatory response in the brain is generated by microglial cells followed by the excessive release of cytotoxic factors, that are negatively affecting the normal function of the brain. Thus, rendering them an important co-factor in the pathogenesis of various neurodegenerative disorders [13]. Microglia cells are localized throughout the central nervous system and constitute 15% of all brain cells. They act as macrophages, that play a significant role in immunoresponse and homeostasis of the brain, recognizing bacterial and viral molecules, tissue damage, protein aggregates, and macromolecules that are released during cell apoptosis. Moreover, the microglia is responsible for synapse plasticity. However, as the brain ages, microglia cells undergo a series of structural modifications, leading to prolonged activation and the establishment of a chronic inflammatory condition, interlinked with oxidative stress [14,15,16,17].

Cholesterol plays a pivotal role as a promoter of inflammation, both in the periphery and in the central nervous system. It is considered a molecular activator of inflammation. In healthy conditions, cholesterol acts as an essential biomembrane ingredient, a membrane viscosity regulator, and a steroid biosynthesis lead molecule. The brain has a high cholesterol concentration, which contributes significantly to neuron plasticity. However, increased levels of cholesterol have been correlated with various conditions, such as cardiovascular diseases, diabetes, metabolic syndrome, and neurodegeneration [18,19].

**Figure 1:**
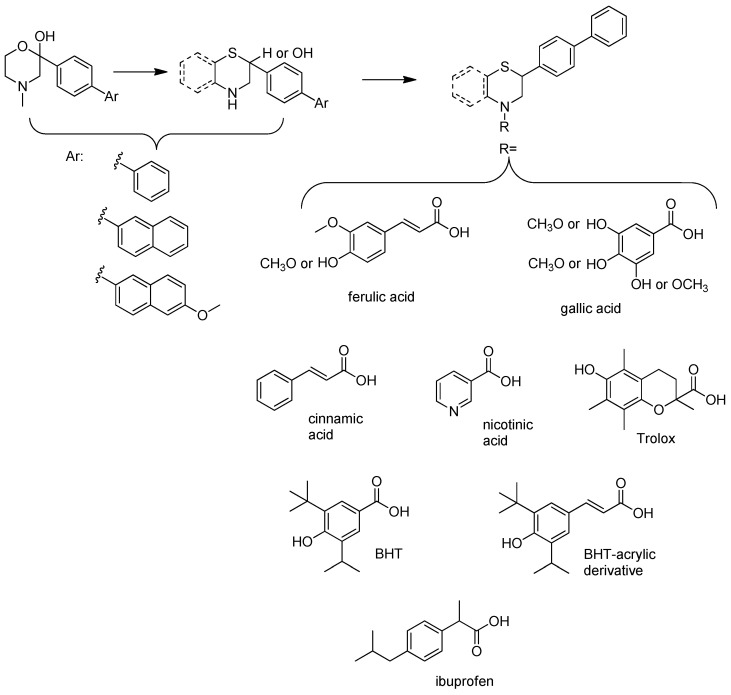
The lead molecules/moieties used for the design of the core structures of this study.

Thus, multitarget agents aiming at all the above-mentioned parameters may serve as a promising approach against the multifactorial nature of neurodegeneration. In this study, molecules with multiple activities on the above-mentioned axes were structurally combined to facilitate new derivatives, bearing two or more properties with the potential of tackling parameters such as oxidative stress, (neuro)inflammation, and lipid dysregulation. Based on our previously developed compounds [20,21,22,23,24,25], with strong antihyperlipidaemic activity, in this study two new core structures were designed which were combined with moieties with known antioxidant and/or anti-inflammatory properties (i.e., phenolic acids and derivatives, a hydrophilic analog of antioxidant Vitamin E, nicotinic acid, and the non-steroidal anti-inflammatory drug ibuprofen). Indeed, phenolic acids have a wide range of biological and therapeutic applications, including neurodegeneration. Especially ferulic acid, cinnamic acid, gallic acid, and caffeic acid are known as promising building blocks for neuroprotection, while playing a catalytic role in anti-apoptotic mediators, cellular proliferation, (neuro)inflammation, cellular signaling, immune response, memory, and learning [26,27,28]. Nicotinic acid also plays an important role in protection against neuroinflammation, mainly via restoring lipid homeostasis in the central nervous system (CNS) [29]. Trolox, the hydrophilic analog of Vitamin E, is characterized by its strong antioxidant activity, as well as cellular protection against neurodegeneration and Alzheimer’s disease [30]. Finally, butylated hydroxytoluene (BHT) and its analogs are also known for their strong antioxidant and anti-inflammatory activity profile [31]. The lead molecules for the design of the core structures of this study, as well as the antioxidant/anti-inflammatory moieties used in the newly designed structures, are shown in Figure 1.

## 2. Results and Discussion

### 2.1. Synthesis

The newly designed compounds **1**–**17** (structures in Table 1, also presented in Supplementary Materials in Table S1) were synthesized via synthetic routes of 3 to 7 steps, at satisfactory yields (>50%) for most steps. The core structures of thiomorpholine (c) and benzothiomorpholine (**11**) derived from the cyclization of mercaptoethylamine or aminothiophenol with the corresponding brominated acid and reduction in the derived lactam ring (Figure 1).

**Figure 2:**
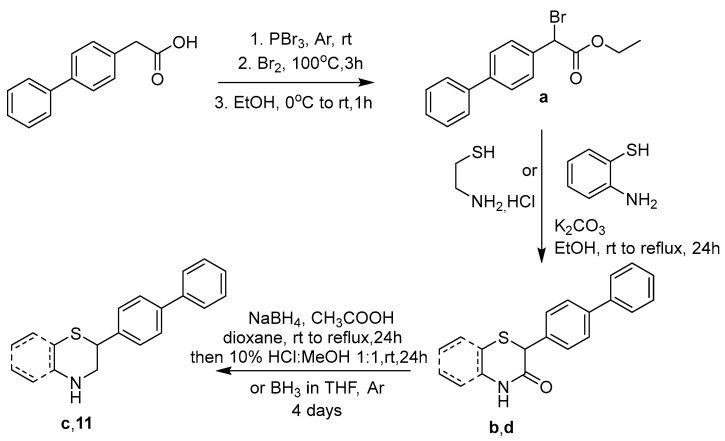
Synthetic route for the core thiomorpholine (**c**) and benzothiomorpholine (**11**) structures.

**Figure 3:**
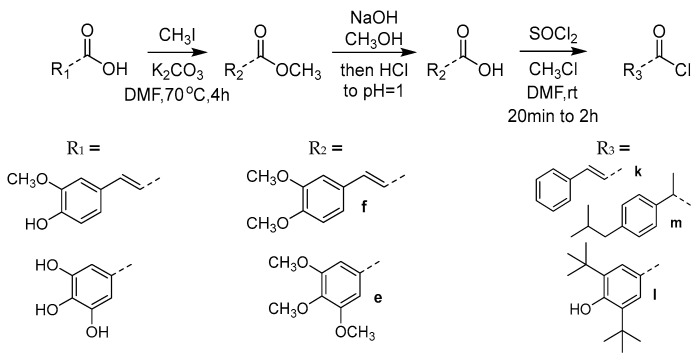
Synthetic route for intermediate acylchloride structures.

**Figure 4:**
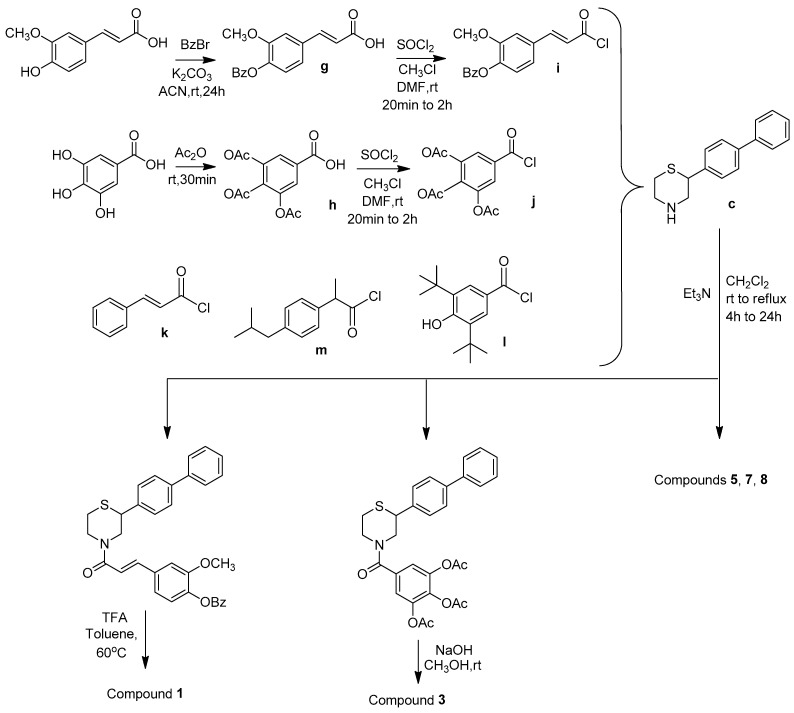
Synthetic route for compounds **1**, **3**, **5**, **7,** and **8**.

**Figure 5:**
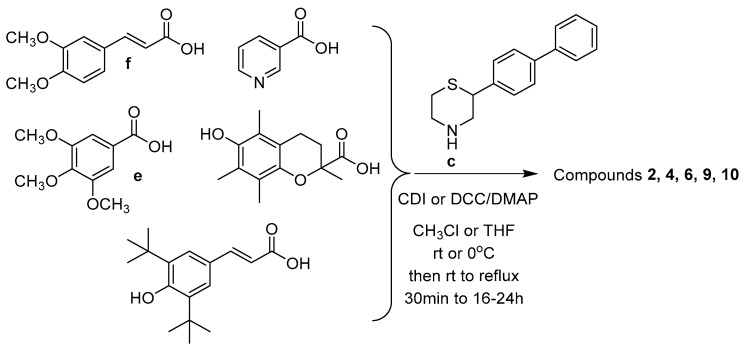
Synthetic route for compounds **2**, **4**, **6**, **9,** and **10**.

**Figure 6:**
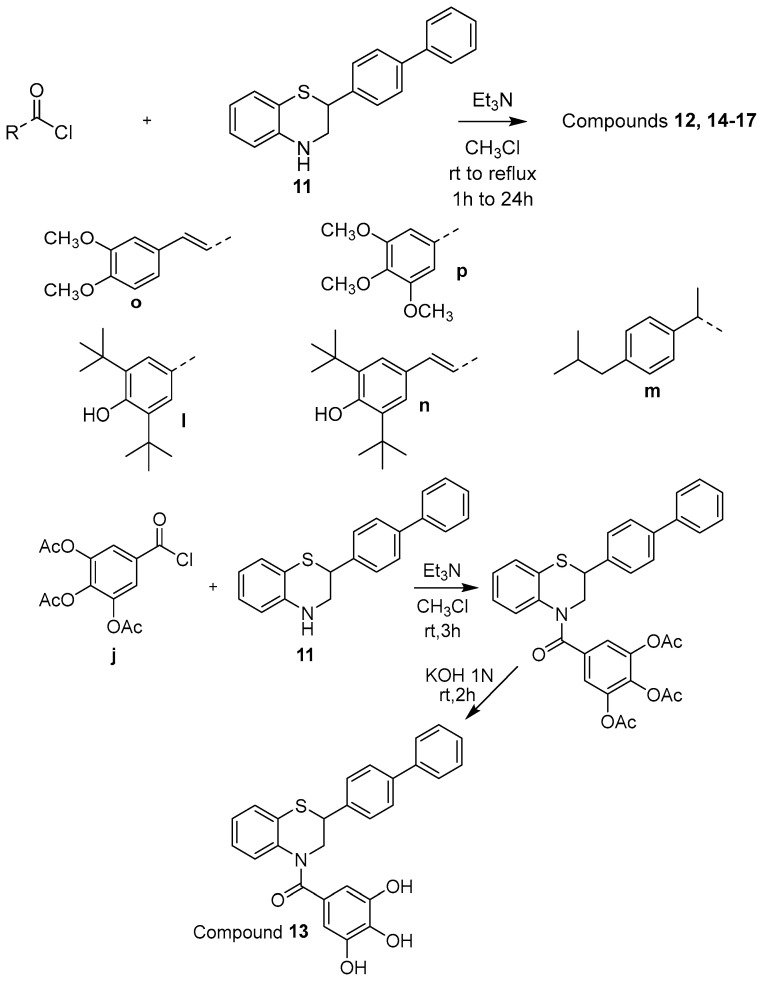
Synthetic route for compounds **12–17**.

The phenolic acid methylated derivatives were derived from the use of iodomethane on the corresponding phenolic acid, whereas protection of benzyl or acetyl groups were employed in specific cases of hydroxyl groups, to avoid byproducts during the formation of the final compounds. The newly designed molecules were derived from the formation of an amide bond between the nitrogen of the core structures and the carboxylic group of each substituent, either via the formation of a carboxyl-chloride or via the use of the coupling reagents 1,1′-carbonyldiimidazole (CDI) and N,N’-dicyclohexylcarbodiimide (DCC) (Figure 2, Figure 3, Figure 4, Figure 5 and Figure 6).

### 2.2. Evaluation of Pharmacological Activity of the Novel Compounds

#### 2.2.1. Anti-Inflammatory Profile

**Figure 7:**
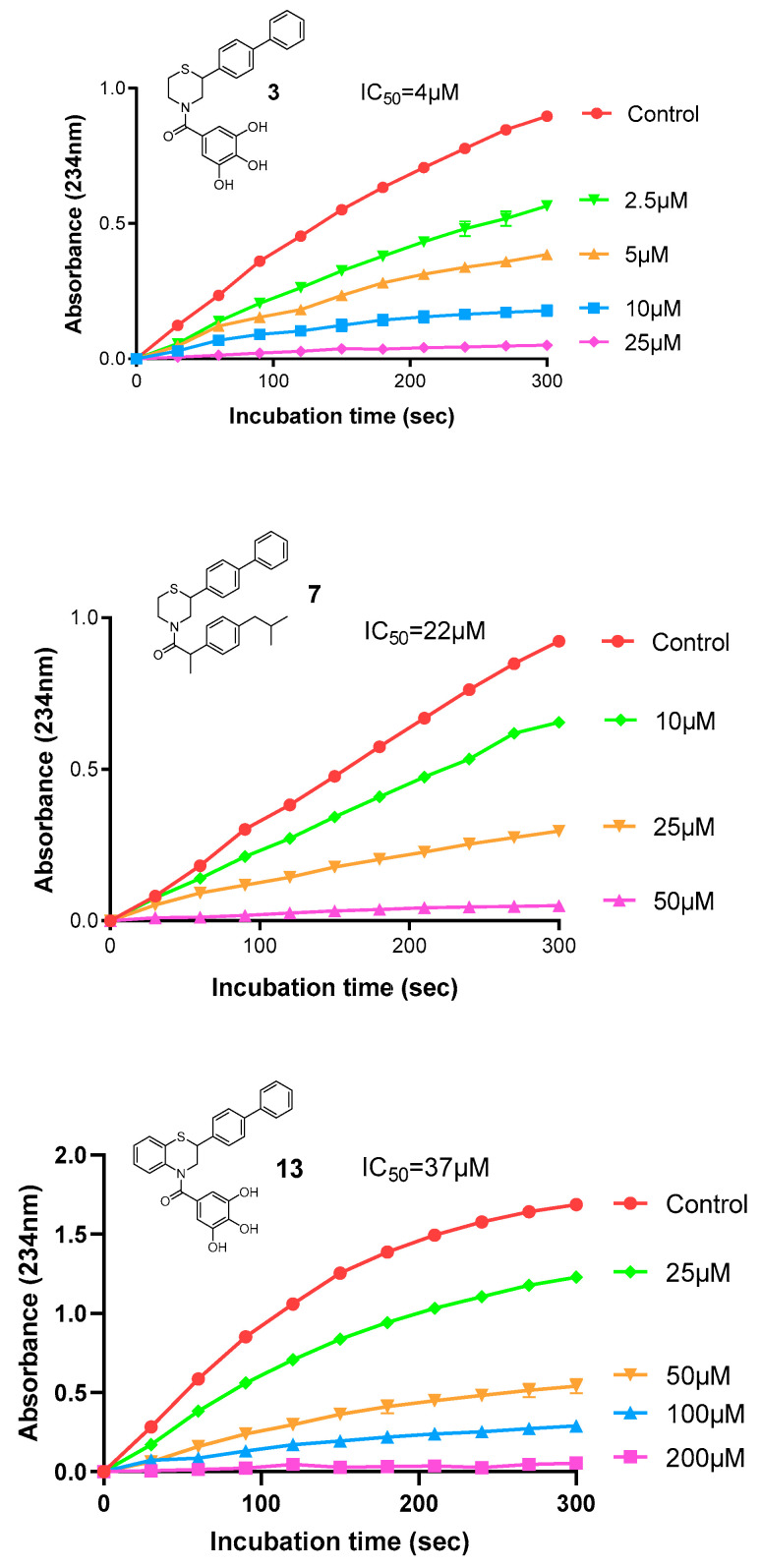
Representative graphs showing the time-dependent inhibition of LOX-3 by different concentrations of derivative **3**, **7**, and **13**.

The anti-inflammatory activity of compounds **1**–**17** was tested via their ability to inhibit the enzyme soybean lipoxygenase (LOX-3). Various concentrations of each derivative were tested and the results are expressed as IC_50_ values. Compound **3** (IC_50_ = 4 μΜ) was the most active of the series, followed by derivatives **7** (IC_50_ = 22 μΜ), **13** (IC_50_ = 37 μΜ), **16** (IC_50_ = 58 μΜ), **1** (IC_50_ = 68 μΜ), **4** (IC_50_ = 71 μΜ), **14** (IC_50_ = 71 μΜ), **12** (IC_50_ = 74 μΜ), and **15** (IC_50_ = 83 μΜ), while the rest of the compounds had IC_50_ values greater than 100 μΜ. Comparing these results with the IC_50_ values of the reference compounds cinnamic acid (>300 μM), ferulic acid (132 μM), ibuprofen (>300 μM), and naproxen (25 μM), it becomes obvious that the newly designed compounds bear important anti-inflammatory activity (via their inhibition of LOX) that has been incorporated by the moieties added on the main (benzo)thiomorpholine structure (**11**), mainly due to the presence of hydrogen bond acceptors/donors and phenolic hydroxylic groups, which enhance the possibility for further interactions with the enzyme’s active site. The IC_50_ values for the tested compounds, as well as representative graphs of the most active, are shown in Table 2 and Figure 7.

Soybean lipoxygenase (LOX-3) corresponds well with human 15-LOX, an enzyme largely correlated with various inflammatory pathways. Moreover, it has now emerged as a possible anti-neurodegenerative molecular target, due to its inductive effect on tau hyperphosphorylations and Aβ formation [32,33].

**Figure 8:**
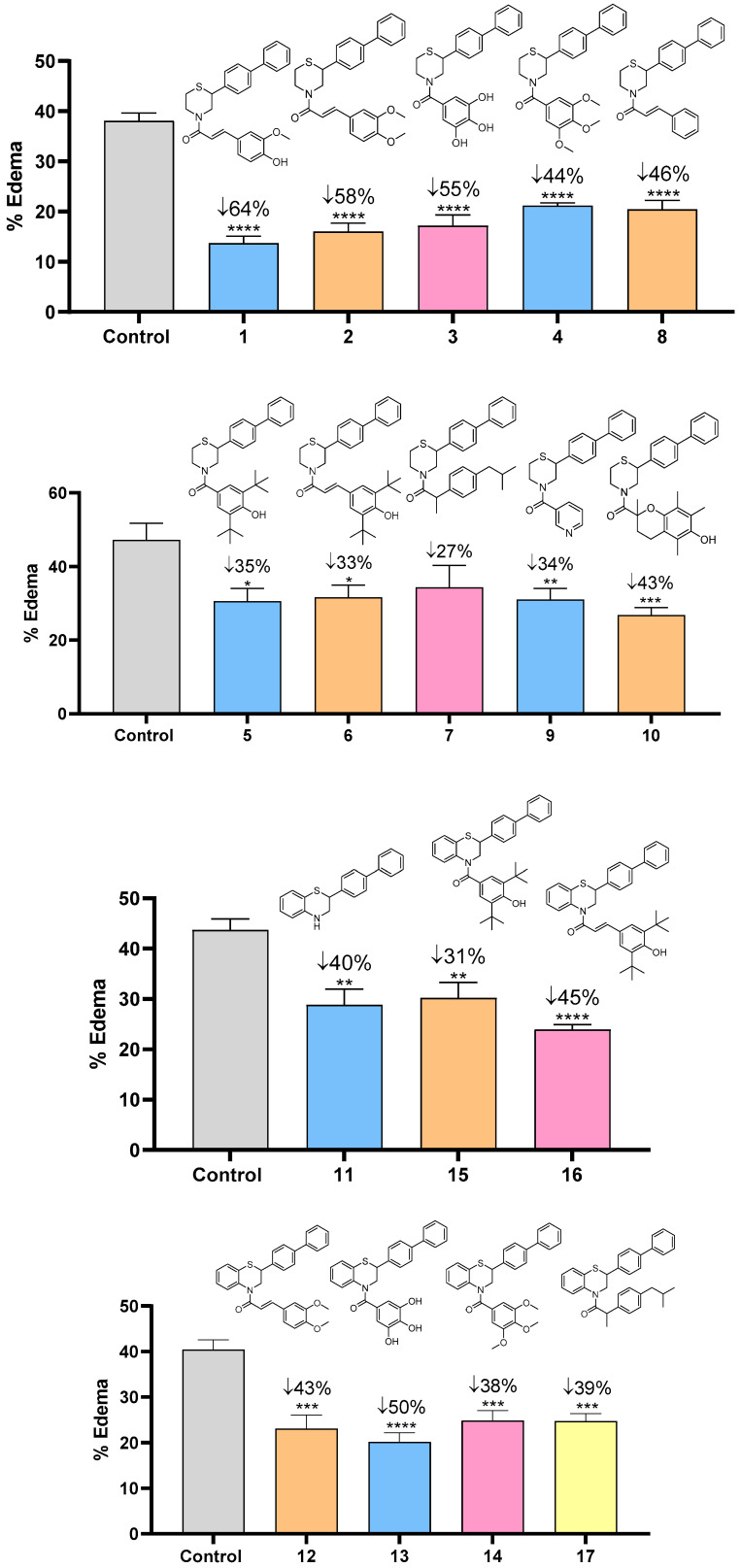
Percentage (%) of carrageenan-induced mouse paw edema in female C57BL/6, after i.p. administration of the tested compounds **1**–**17**, at a dose of 0.30 mmol/kg of body weight, compared to control group. Each value represents the mean obtained from six animals. Significant difference from carrageenan-treated control: * *p* < 0.05, ** *p* < 0.01, *** *p* < 0.001, **** *p* < 0.0001.

The anti-inflammatory activity was further tested in vivo, via the ability of the novel compounds to inhibit carrageenan-induced mouse paw edema. The results are shown in the next graphs (Figure 8). Almost all compounds inhibited edema by more than 40%, with the most active ones being **1**, **2**, **3,** and **13**, bearing the anti-inflammatory moiety of ferulic acid (**1**), methylated ferulic acid, (**2**) and gallic acid (**3** and **13**), reaching up to 60% inhibition of mouse paw edema.

#### 2.2.2. Antioxidant Profile

Compounds **1**–**17** were evaluated for their ability to scavenge the free radical DPPH. Among the thiomorpholine derivatives **1**–**10**, compounds **3** and **10** were the most potent, presenting IC_50_ values of 31 μM and 78 μM, respectively (Table 3). Compound **1** also showed moderate scavenging activity, bearing an IC_50_ value of 166 μM. Among the benzothiomorpholine derivatives **11**–**17**, compounds **13** and **16** were the most potent, with IC_50_ values of 36 μM and 141 μM, respectively. Regarding the parent molecules that could be considered here as reference antioxidants, the most active ones proved to be Trolox (IC_50_ = 33 μM), gallic acid (IC_50_ = 14 μM), BHT, and its acrylic derivative (IC_50_ = 31 μM and 77 μM, respectively) which maintained their effectiveness when incorporated in the novel multitarget compounds (Table 1). An important factor for ensuring scavenging capacity is the presence of free phenolic hydroxyl groups, that seem to stabilize the free radicals.

**Figure 9:**
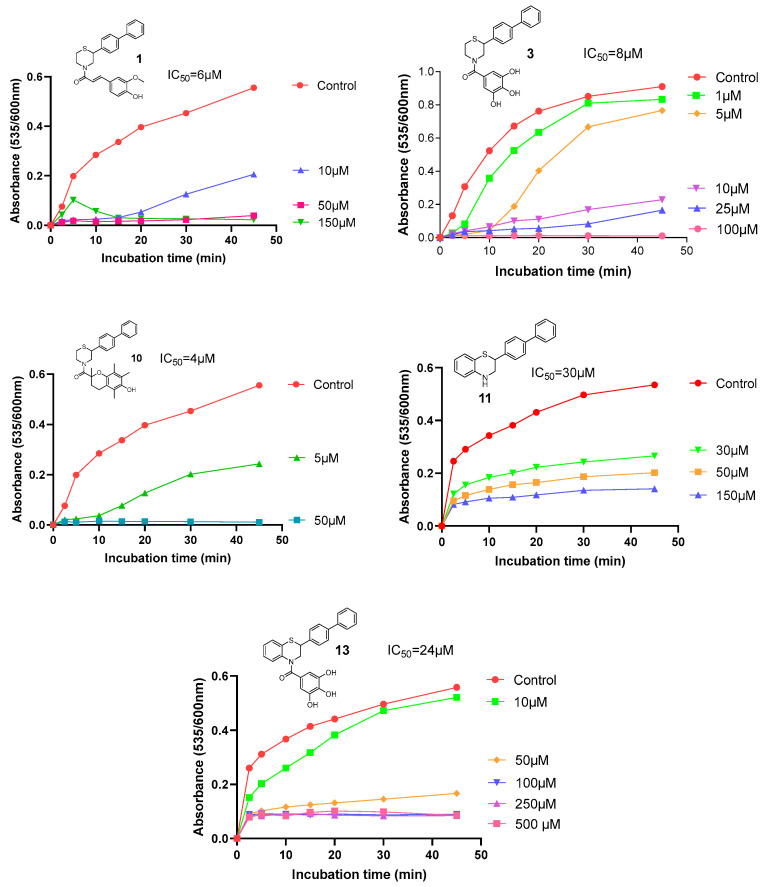
Representative graphs showing the time-dependent inhibition of lipid peroxidation by different concentrations of the most active compounds **1**, **3**, **10**, **11,** and **13**.

The antioxidant effect of the new compounds was also evaluated via their ability to protect against mouse liver microsomal lipid peroxidation (LP). Results were expressed as IC_50_ values after a 45 min incubation period (Table 3) [24,34]. Again, the compounds bearing the highest protective activity against lipid peroxidation, with IC_50_ values below 10 μM, were compounds **10** (IC_50_ = 4 μM), **1** (IC_50_ = 6 μM), and **3** (IC_50_ = 8 μM), followed by compounds **13** (IC_50_ = 24 μM) and **11** (IC_50_ = 30 μM). Compared to reference compounds, Trolox (43% inhibition at 100 μM) and gallic acid (73% inhibition at 100 μM) were the most active, while the new derivatives have comparably high activity. Representative graphs of the most active molecules are shown in Figure 9. The highest activity correlates with the presence of the moieties 6-hydroxy-2,5,7,8-tetramethylchroman-2-carboxylic acid, ferulic acid, and gallic acid, bearing methyl and hydroxyl groups on benzyl ring.

Another attribute evaluated, which may contribute to the antioxidant capacity of the newly designed molecules, was their ability to chelate iron ions (via the ferrozine assay). A solution of each derivative was incubated with a solution of iron ions and then the triazine ferrozine was added, to react with the remaining unchelated ions. The results are expressed as percentage of chelation for the corresponding concentration (Table 3). The most active derivatives were the gallic acid analogs **3** and **13**, which chelated iron ions by 89% and 91%, respectively, at a concentration of 100μM, while they showed important chelating ability even at a low concentration of 10μM. Free and methylated phenol groups are crucial components for chelation, increasing the potency of the derivatives bearing these groups. The evaluation of the chelating activity of the parent compounds showed that the most active were gallic acid and Trolox, chelating iron ions by 98% and 29%, respectively, at a concentration of 100μM.

In total, the in vitro antioxidant profile of the novel molecules was moderate, apart from the significant results in the lipid peroxidation assay. However, the in vivo assessment of the possible antioxidant activity of all novel molecules showed impressive results for most of the compounds. More specifically, 12 out of the 17 derivatives increased total antioxidant capacity (TAC) of the plasma in hyperlipidaemic mice by more than 100%. The respective results are shown in Table 4. The most active derivatives were **1**, **3**, **6**, **10**, **11,** and **16**, which increased TAC by more than 200%, while compounds **3** and **6** caused an increase of almost 800%. Compounds **1**, **3**, and **10** are quite effective antioxidants in vitro and maintained or even enhanced this profile in vivo. The superior in vivo antioxidant profile of the derivatives could possibly be attributed to favorable pharmacodynamic/pharmacokinetic aspects: multitarget agents are more likely to be more beneficial in more complex procedures, i.e., those that involve more than one factor, as in in vivo conditions. An antioxidant profile is not always restricted to one specific mechanism, e.g., radical scavenging. On the contrary, such complex molecular mechanisms are connected to various pathways present in the whole organism, and thus only in vivo assays can properly expose the multifunctional activity of such agents. This fact is supported by the important activity of compounds **11** and **16**, which showed no significant antioxidant activity in vitro, while having an excellent activity profile in vivo.

#### 2.2.3. Inhibitory Activity Against Acetylcholinesterase (AChE)

Another known molecular target correlated with neurodegeneration is the activity of the enzyme acetylcholinesterase (AChE). The potential inhibitory activity against this enzyme was evaluated and the results are expressed as percentage (%) of inhibition at the tested concentration (300 μM) (Table 5). Most of the derivatives showed moderate activity, inhibiting AChE by 10–33%. However, compounds **6**, **10**, **13,** and **14** showed satisfactory inhibitory activity against AChE, inhibiting it by approximately 45%. Given the inactivity of the core thiomorpholine and benzothiomorpholine structure (compound **11**, Table 5), the improved inhibitory capacity of the derivatives is a result of the optimization of their substitution, geometry, and lipophilicity (larger and more lipophilic molecules), maximizing their lipophilic interaction with the enzyme’s active center. The increased number of heteroatoms, which act as hydrogen bond acceptors/donors as well as the phenolic hydroxyl groups incorporated in the core structures are important factors towards this direction. The addition of the phenolic acids and their methylated derivatives, as well as known antioxidant moieties, such as 6-hydroxy-2,5,7,8-tetramethylchroman, led to a significant increase in molecular size and optimal geometry of the novel compounds, possibly resulting in higher enzyme–ligand interactions. This modification of the physicochemical profile of the newly designed derivatives led to the most active molecules **6** (43% inhibition), **10** (45% inhibition), **13** (43% inhibition), and **14** (47% inhibition).

It should be mentioned that many natural polyphenolic acids and alkaloids with IC_50_ values in the range of 100–200 µM against AChE are commonly treated as viable leads for optimization, despite their modest in vitro potency [35,36,37]. Classic examples include chlorogenic acid (IC_50_ approx. 277 µM) and syringic acid (IC_50_ approx. 149 µM). Moreover, hydroxybenzoic and hydroxycinnamic acids such as vanillic, caffeic, and ferulic acid often display weak-to-moderate AChE inhibition in the range of tens to hundreds of micromolar. Especially ferulic acid derivatives can be optimized to achieve much stronger inhibition. These data indicate that compounds with IC_50_ values in the 100–200 µM range should not be disregarded, as they provide structurally diverse, safe, and accessible scaffolds that can serve as starting points for multitarget-directed ligand development and subsequent medicinal chemistry optimization. Apart from polyphenolic acids, ibuprofen and some of its derivatives have also been studied for their effect on longevity and geroprotective action in *Drosophila* spp., while pyridine, quinoxaline, and s-triazine derivatives have been shown to combine antioxidant and AChE inhibitory activity [38,39].

#### 2.2.4. Antihyperlipidaemic Profile of the Novel Compounds

The core structures of thiomorpholine and benzothiomorpholine were designed according to molecules previously developed and studied by our laboratory, as inhibitors of the enzyme squalene synthase, an enzyme involved in cholesterol biosynthesis [21,22,23,25]. Thus, the novel compounds were assessed for their antihyperlipidaemic activity, in an in vivo hyperlipidemia protocol, induced by the administration of tyloxapol in male C25BL/6 mice [21,22,23,25]. Lipidaemic parameters, such as total cholesterol (TC), triglycerides (TG), and high-density lipoprotein (HDL) were measured in the plasma of the different groups and compared to the tyloxapol-treated control group. The parameters of low-density lipoprotein (LDL), as well as the antiatherogenic index (HDL/LDL), were calculated accordingly. The results are summarized in Table 6.

According to the results, the novel compounds present a strong antihyperlipidaemic profile. More than half of the derivatives reduced TC levels by more than 40%, whereas almost all compounds caused a significant decrease in TG levels by more than 50% and up to 86%. Apart from the decrease in these parameters, the novel derivatives improved the lipidaemic profile of the treated mice, by increasing HDL and decreasing LDL levels. Indeed, more than 80% of the tested compounds increased HDL levels by up to 130%, while more than 90% of the tested compounds decreased LDL levels by up to 80%. This improvement in the lipidaemic profile is also confirmed via their antiatherogenic index, expressed as the ratio HDL/LDL. Indeed, the new derivatives not only increased the antiatherogenic index, but this increase was greater than 100% for most of the compounds. In particular, for compounds **1**, **3,** and **11**–**17**, the decrease was greater than 200%. The compounds with the most impressive total antihyperlipidaemic activity were **3**, **15,** and **16**, which increased the antiatherogenic index of the mice by more than 600%. This in vivo antidyslipidaemic profile is attributed to their possible interaction with the cholesterol biosynthesis enzyme squalene synthase, since they structurally resemble other such known inhibitors [21,22,23,25].

### 2.3. Physicochemical Properties of the Novel Compounds

Various physicochemical parameters were calculated, along with the potential of the new derivatives to cross the blood–brain barrier (BBB). Parameters, such as molecular weight, lipophilicity, number of hydrogen bond donors and/or acceptors, as well as polar surface area of the drug candidate significantly affect its potential ability to cross through lipid membranes. The potential crossing through the blood–brain barrier was calculated for each new derivative, using an equation in ref. [40], which results in a score for logBB. If this score is above 0.3, the compound is characterized as CNS+ (central nervous system-positive), bearing high potential for BBB permeability. As shown in Table 7, almost 90% of the new compounds were characterized as CNS+, according to their calculated logBB, rendering them promising candidates for expressing their properties and activities inside the CNS.

## 3. Materials and Methods

### 3.1. Chemical Synthesis

#### 3.1.1. Ethyl 2-([1,1′-Biphenyl]-4-yl)-2-bromoacetate (**a**)

In 7.07 mmol of 4-biphenylacetic acid, 17.67 mmol of phosphorus tribromide is added at room temperature, under inert atmosphere. After stirring for 3 h, bromine is added and the reaction mixture is heated at 100 °C under Argon for 3 h. Afterwards, dry ethanol is added dropwise, under cooling (0 °C). The reaction is continued for 30 min at 0 °C and for another 30 min at room temperature. Finally, the reaction is diluted with diethyl ether, the organic phase is washed with water, sodium metabisulfite 5%, saturated solution of sodium bicarbonate, and sodium chloride, respectively, dried over sodium sulfate and evaporated to dryness. The product is used without further purification in the next step. Yield: 100%, off-white oil [24,25]. ^1^H NMR (400 MHz, CDCl_3_) δ (ppm): 1.25 (t, *J* = 7.14 Hz, 3H, -OCH_2_CH_3_), 4.20 (q, *J* = 7.24 Hz, 2H, -OCH_2_CH_3_), 5.33 (s, 1H, -CHBr), 7.29–7.34 (m, 1H, 4′-H biphenyl), 7.37–7.42 (m, 2H, 3,5-H biphenyl), 7.51–7.58 (m, 6H, 2,6,3′,5′,2′,6′-H biphenyl).

#### 3.1.2. 2-([1,1′-Biphenyl]-4-yl)thiomorpholine-3-one (**b**)

To a mixture of 14.85 mmol potassium carbonate in ethanol, 7.07 mmol of **a** is added under stirring. Afterwards, 8.48 mmol of mercaptoethylamine hydrochloride is added and the mixture is heated under reflux for 24 h. Finally, the mixture is cooled at room temperature and diluted with cold water, to facilitate the precipitation of a white solid. The mixture is filtrated, washed with ethyl acetate and used in the next step. Yield: 99%, white solid. M.p.: 221.0–223.0 °C [25]. ^1^H NMR (400 MHz, CDCl_3_) δ (ppm): 2.86 (brs, 2H, 6-CH_2_), 3.64 (brs, 2H, 5-CH_2_), 4.61 (s, 1H, 2-CH_2_), 6.56 (s, 1H, 4′-H biphenyl), 7.20 (d, *J* = 8.31 Hz, 2H, 3,5-H biphenyl), 7.37 (d, *J* = 7.09 Hz, 2H, 2′,6′-H biphenyl), 7.42–7.49 (m, 4H, 2,6,3′,5′-H biphenyl), 7.66 (s, 1H, -NH).

#### 3.1.3. 2-([1,1′-Biphenyl]-4-yl)thiomorpholine (**c**)

To a dry flask, 0.93 mmol of **b** in dioxan is added under cooling (0 °C), followed by 6.96 mmol of sodium borohydride and 6.96 mmol of acetic acid. The reaction mixture is stirred at room temperature for 30 min and at reflux for 24 h. Afterwards, the mixture is cooled at room temperature, diluted with cold water, and stirred for another hour. Finally, a mixture of 10% hydrochloric acid/methanol at a ratio of 1:1 is added and stirred for 24 h. Dioxane is evaporated and the aqueous phase is extracted with ethyl acetate, dried over sodium sulfate and evaporated. The product is isolated via flash chromatography. Yield: 62%, off-white solid. M.p.: 140.0–141.0 °C [25]. ^1^H NMR (400 MHz, CDCl_3_) δ: 1.83 (s, 1H, NH), 2.51–2.56 (m, 1H, 6-Hax), 2.87–2.98 (m, 2H, 6-Heq και 5-Hax), 3.03–3.12 (m, 1H, 5-Heq), 3.23–3.36 (m, 2H, 3-CH_2_), 3.84–3.93 (m, 1H, 2-Hax), 7.16–7.20 (m, 2H, 2,6-H biphenyl), 7.27–7.29 (m, 1H, 4′-H biphenyl), 7.36 (d, *J* = 8.60 Hz, 2H, 2′,6′-H biphenyl), 7.43–7.49 (m, 3H, 3′,5′,3-H biphenyl), 7.60 (ds, *J* = 1.76 Hz, 1H, 5-H biphenyl).

#### 3.1.4. 2-([1,1′-Biphenyl]-4-yl)-2H-benzo[b][1,4]thiazin-3(4H)-one (**d**)

To a dry flask, 10.6 mmol of 2-aminothiophenol is dissolved in dry dimethylformamide under cooling (0 °C). Then, 7.07 mmol of **a** is added and stirred at this temperature for 30 min and at room temperature for 2 days. Afterwards, the mixture is extracted with dichloromethane and the organic phase is washed with water and a saturated solution of sodium chloride. The organic phase is evaporated and ethyl acetate is added to the residual, facilitating the precipitation of the desired product, which is used in the next step. Yield: 89%, white solid. M.p.: 235.0–236.5 °C [24]. ^1^H NMR (400 MHz, CDCl_3_) δ (ppm): 4.68 (s, 1H, 2-CH benzothiazine), 6.81 (d, *J* = 8.02 Hz, 1H, 5-CH benzothiazine), 6.96 (dt, *J*_1_ = 1.17 Hz, *J*_2_ = 7.62 Hz, 1H, 7-CH benzothiazine), 7.11 (dt, *J*_1_ = 1.18 Hz, *J*_2_ = 7.83 Hz, 1H, 6-CH benzothiazine), 7.25–7.29 (m, 2H, 8-CH benzothiazine και 4′-CH biphenyl), 7.34 (d, *J* = 7.83 Hz, 2H, 3,5-CH biphenyl), 7.38 (d, *J* = 8.22 Hz, 2H, 3′,5′-CH biphenyl), 7.45–7.48 (m, 4H, 2,6,2′,6′-CH biphenyl), 8.55 (s, 1H, NH).

#### 3.1.5. General Synthesis of Compounds **e**, **f**

To a dry flask, 1.76 mmol of gallic acid (for **e**) or 1.54 mmol of (E)-ferulic acid (for **f**) are dissolved in dimethylformamide. Then, 7.7 mmol of potassium carbonate and 15.4 mmol iodomethane are added. The reaction mixture is heated at 70 °C for 4 h. Afterwards, it is filtered and the filtrate is extracted with dichloromethane, dried over sodium sulfate and evaporated to isolate the intermediated methylesters of the respective acids. Afterwards, 1.76 or 2.00 mmol of each methylester (for **e** or **f**, respectively) is dissolved in methanol and 17.6 mmol of sodium hydroxide in methanol/water at a ratio of 9:1 is added. Finally, the reaction mixture is acidified with hydrochloric acid to pH = 1, to facilitate the precipitation of the desired products.

##### 3,4,5-trimethoxybenzoic Acid (**e**)

Yield: 69%, white solid. M.p.: 169.0 °C [41]. ^1^H NMR (400 MHz, CDCl_3_) δ (ppm): 3.93 (s, 6H, 3,5-OCH_3_), 3.94 (s, 3H, 4-OCH_3_), 7.38 (s, 2H, 2,6-aromatic).

##### (*E*)-3-(3,4-dimethoxyphenyl)acrylic Acid (**f**)

Yield: 78%, M.p.: 189.0–191.0 [42]. ^1^H NMR (400 MHz, CDCl_3_) δ: 3.93 (s, 6H, 3,4-OCH_3_), 6.33 (d, *J* = 15.9 Hz, 1H, 3-CH), 6.88 (d, *J* = 8.3 Hz, 1H, aromatic), 7.08 (d, *J* = 1.9 Hz, 1H, aromatic), 7.14 (dd, *J* = 8.3, 1.9 Hz, 1H, aromatic) 7.74 (d, *J* = 15.9 Hz, 1H, 2-CH).

#### 3.1.6. (*E*)-3-(4-(Benzyloxy)-3-methoxyphenyl)acrylic Acid (**g**)

To a dry flask, 1.00 mmol (*E)*-3-(4-hydroxy-3-methoxyphenyl)acrylic acid is dissolved in acetonitrile and 2.00 mmol potassium carbonate as well as 2.5 mmol benzylbromide are added. The reaction mixture is stirred at room temperature for 24 h and then hydrolyzed with sodium hydroxide in methanol/water at a ratio of 9:1, to facilitate the isolation of the desired product. Yield: 70%, white solid, M.p.: 191.0–192.0 [40]. ^1^H NMR (400 MHz, CDCl_3_) δ (ppm): 3.80 (s, 3H, -OCH_3_), 5.19 (s, 2H, -OCH_2_-), 6.30 (d, *J* = 15.9 Hz, 1H, 3-CH), 6.87 (d, *J* = 8.3 Hz, 1H, phenyl), 7.03 (dd, *J* = 8.5, 2.0 Hz, 1H, phenyl), 7.07 (d, *J* = 2.0 Hz, 1H, phenyl), 7.29–7.33 (m, 1H, benzyl), 7.37 (ddd, *J* = 7.4, 4.5, 1.3 Hz, 2H, benzyl), 7.41–7.45 (m, 2H, benzyl), 7.62 (d, *J* = 15.9 Hz, 1H, 2-CH).

#### 3.1.7. 3,4,5-Triacetoxybenzoic Acid (**h**)

To a dry flask, 1.18 mmol gallic acid and 0.68 mL acetic anhydride are added. The reaction mixture is stirred at room temperature for 30 min. Afterwards, the reaction is diluted with water, extracted with ethyl acetate, and the organic phase is dried over sodium sulfate and evaporated. Yield: 100%, white solid. M.p.: 172.0 °C [43]. ^1^H NMR (400 MHz, CDCl_3_) δ (ppm): 2.308 (s, 6H, 3,5-CH_3_COO-), 2.314 (s, 3H, 4-CH_3_COO-), 7.85 (s, 2H, aromatic).

#### 3.1.8. General Synthesis of Compounds **i**–**m**

To a dry flask, 1.0 mmol of the respective acid **g**, **h**, trans-cinnamic acid, 3,5-ditertbutyl-4-hydroxy-benzoic acid, 2-(4-isobutylphenyl)propanoic acid, (*E*)-3-(3,4-dimethoxyphenyl)acrylic acid, 3,4,5-trimethoxybenzoic acid, or (*E*)-3-(3,5-ditertbutyl-4-hydroxyphenyl)acrylic acid is dissolved in chloroform and 4.0 mmol of thionylchloride is added, together with 2–5 drops of dimethylformamide. The reaction mixture is stirred at room temperature for 20 min to 2 h. Afterwards, the thionylchloride is evaporated and the desired chloride [(*E*)-3-(4-(benzyloxy)-3-methoxyphenyl)acryloyl chloride (**i**), 3,4,5-triacetoxybenzoyl chloride (**j**), trans-cinnamoyl chloride (**k**), 3,5-ditertbutyl-4-hydroxybenzoyl chloride (**l**), 2-(4-isobutylphenyl)propanoyl chloride (**m**), (*E*)-3-(3,5-ditertbutyl-4-hydroxyphenyl)acryloyl chloride (**n**), (*E*)-3-(3,4-dimethoxyphenyl)acryloyl chloride (**o**), or 3,4,5-trimethoxybenzyloyl chloride (**p**)] (as a yellow oil in all cases) is used in the next step without further purification.

#### 3.1.9. General Synthesis of Compounds **1**, **3**, **5**, **7,** and **8**

To a dry flask, 1.0 mmol of **c** is dispersed in dichloromethane and 1.5 mmol triethylamine is added. Then, 1.0 mmol of the respective chloride, **i**–**m**, is added dropwise, dissolved in chloroform. The reaction mixture is stirred at room temperature or at reflux for 4–24 h. Afterwards, it is diluted with chloroform and the organic phase is washed with water, dried over sodium sulfate and evaporated. The desired products are isolated with flash chromatography. Regarding compounds **1** and **3**, another step is required to deprotect the benzoyl- and acetyl- groups, by treatment with potassium hydroxide 1N in methanol (at room temperature) and trifluoroacetic acid in toluene (at 60 °C), respectively.

##### (*E*)-1-(2-([1,1′-Biphenyl]-4-yl)thiomorpholino)-3-(4-hydroxy-3-methoxyphenyl)prop-2-en-1-one (**1**)

Total yield: 57%, yellow solid. M.p.: 98.6–99.7 °C. ^1^H NMR (400 MHz, CDCl_3_) δ (ppm): 1.69 (s, 2H, 6-CH_2_ thiomorpholine), 2.71 (d, *J* = 13.8 Hz, 1H, 5-CH_2_ thiomorpholine), 2.99 (t, 1H, 5-CH_2_ thiomorpholine), 3.20 (s, 1H, 3-CH_2_ thiomorpholine), 3.45–3.70 (m, 1H, 3-CH_2_ thiomorpholine), 3.93 (s, 3H, -OCH_3_), 3.98 (s, 1H, 2-CH thiomorpholine), 6.93 (t, *J* = 6.7 Hz, 2H, phenyl), 6.99 (s, 1H, phenyl), 7.11 (d, *J* = 8.1 Hz, 1H, biphenyl), 7.28 (d, *J* = 7.9 Hz, 2H, biphenyl), 7.39 (dd, *J* = 7.9, 1.7 Hz, 1H, biphenyl), 7.42–7.49 (m, 1H, biphenyl), 7.53–7.58 (m, 3H, biphenyl), 7.65 (d, *J* = 15.2 Hz, 2H, 2,3-CH propenone), 7.71 (s, 1H, 5-H phenyl).

^13^C NMR (101 MHz, CDCl_3_) δ (ppm): 15.27 (6-C thiomorpholine), 44.55 (2-C thiomorpholine), 47.83 (5-C thiomorpholine), 56.08 (3-C thiomorpholine), 65.87 (-OCH_3_), 110.10 (2-C phenyl), 114.82 (2-C propenone), 116.03 (5-C phenyl), 122.01 (6-C phenyl), 126.98 (2′,6′-C biphenyl), 127.54 (4′-C biphenyl), 129.05 (2,6-C biphenyl), 130.99 (1-C biphenyl), 131.26 (3,5,3′,5′-C biphenyl), 132.48 (4-C biphenyl), 140.22 (1′-C biphenyl), 141.18 (1-C phenyl), 144.08 (3-C propenone), 146.73 (4-C phenyl), 147.61 (3-C phenyl), 168.40 (C=O).

##### (2-([1,1′-Biphenyl]-4-yl)thiomorpholino)(3,4,5-trihydroxyphenyl)methanone (**3**)

Total yield: 20%, off-white solid. M.p.: 166.1–167.2 °C. ^1^H NMR (400 MHz, DMSO-d_6_) δ (ppm): 2.22–2.33 (m, *J* = 19.0 Hz, 2H, 6-CH_2_ thiomorpholine), 2.73 (s, 1H, 5-CH_2_ thiomorpholine), 2.86–2.92 (m, 2H, 5-CH_2_ thiomorpholine, 3-CH_2_ thiomorpholine), 4.02–4.14 (m, 2H, 3-CH_2_ thiomorpholine, 2-CH thiomorpholine), 6.35 (s, 2H, 2,6-C phenyl), 7.36 (d, *J* = 8.4 Hz, 3H, biphenyl), 7.46 (s, 2H, biphenyl), 7.65 (d, *J* = 8.3 Hz, 4H, biphenyl), 8.50 (s, 1H, 4-OH), 9.12 (s, 2H, 3,5-OH). ^13^C NMR (101 MHz, DMSO-d_6_) δ (ppm): 29.16 (6-C thiomorpholine), 29.48 (2-C thiomorpholine), 52.65 (5-C thiomorpholine), 55.38 (3-C thiomorpholine), 106.71 (2,6-C phenyl), 121.83 (1-C phenyl), 127.43 (1-C biphenyl), 129.27 (2′,6′-C biphenyl), 131.57 (2,6-C biphenyl), 131.84 (3,5,3′,5′-C biphenyl), 131.98 (4′-C biphenyl), 132.28 (4-C biphenyl), 135.18 (4-C phenyl), 139.64 (1′-C biphenyl), 146.21 (3,5-C phenyl), 170.41 (C=O).

##### (2-([1,1′-Biphenyl]-4-yl)thiomorpholino)(3,5-ditertbutyl-4-hydroxyphenyl)methanone (**5**)

Yield: 44%, orange semisolid. ^1^H NMR (400 MHz, CDCl_3_) δ (ppm): 1.38 (s, 9H, 3′′ ή 5′′-C(CH_3_)_3_), 1.50 (s, 9H, 3′′ ή 5′′-C(CH_3_)_3_), 2.41–2.50 (m, 1H, 6-CH_2_ thiomorpholine), 2.60–2.75 (m, 2H, 5-CH_2_ thiomorpholine), 2.88–2.97 (m, 1H, 6-CH_2_ thiomorpholine), 3.28 (ddd, *J* = 23.1, 17.7, 5.8 Hz, 1H, 3-CH_2_ thiomorpholine), 3.47 (dd, *J* = 24.6, 12.0 Hz, 1H, 3-CH_2_ thiomorpholine), 3.65–3.78 (m, 1H, 2-CH thiomorpholine), 7.22–7.26 (m, 1H, biphenyl), 7.35–7.56 (m, 7H, biphenyl), 7.88–7.97 (m, 1H, biphenyl), 8.08 (s, 1H, phenyl), 8.13 (s, 1H, phenyl). ^13^C NMR (101 MHz, CDCl_3_) δ (ppm): 29.70 (6-C thiomorpholine), 30.16 (3,5-C(CH_3_)_3_), 31.44 (3,5-C(CH_3_)_3_), 32.86 (2-C thiomorpholine), 34.33 (5-C thiomorpholine), 35.60 (3-C thiomorpholine), 125.13 (2′,6′-C biphenyl), 125.95 (1-C phenyl), 126.36 (4′-C biphenyl), 126.96 (1-C biphenyl), 131.02 (2,6-C biphenyl), 131.25 (3,5,3′,5′-C biphenyl), 132.49 (2,6-C phenyl), 136.34 (3,5-C phenyl), 136.63 (1′-C biphenyl), 136.65 (4-C biphenyl), 155.79 (4-C phenyl), 166.43 (C=O).

##### 1-(2-([1,1′-Biphenyl]-4-yl)thiomorpholino)-2-(4-isobutylphenyl)propan-1-one (**7**)

Yield: 99%, white semisolid. ^1^H NMR (400 MHz, CDCl_3_) δ (ppm): 0.98 (d, *J* = 6.0 Hz, 6H, -CH(CH_3_)_2_), 1.56 (d, *J* = 6.4 Hz, 3H, -CH(CH_3_)), 1.86–2.0 (m, 1H, -CH(CH_3_)_2_), 2.46–2.62 (m, 2H, -CHCH_2_-), 2.82–3.20 (m, 1H, 6-CH_2_ thiomorpholine), 3.34–3.47 (m, 1H, 5-CH_2_ thiomorpholine), 3.47–3.68 (m, 1H, -CH(CH_3_)), 3.76–3.91 (m, 1H, 3-CH_2_ thiomorpholine), 3.94–4.15 (m, 2H, 5,6-CH_2_ thiomorpholine), 4.27 (d, *J* = 14.2 Hz, 1H, 3-CH_2_ thiomorpholine), 4.86–5.06 (m, 1H, 2-CH thiomorpholine), 7.18 (s, 1H, 4′-H biphenyl), 7.20–7.30 (m, 4H, 2,3,5,6-H phenyl), 7.32–7.48 (m, 4H, 2,6,2′,6′-H biphenyl), 7.56–7.72 (m, 3H, 3,3′,5′-H biphenyl), 7.75 (s, 1H, 5-H biphenyl). ^13^C NMR (101 MHz, CDCl_3_) δ (ppm): 20.70 (-CH(CH_3_)), 22.31 (2 × -CH(CH_3_)_2_), 30.11 (-CH(CH_3_)_2_), 35.49 (6-C thiomorpholine), 37.46 (-CH(CH_3_)), 43.03 (2-C thiomorpholine), 43.54 (5-C thiomorpholine), 44.92 (-CHCH_2_-), 57.99 (3-C thiomorpholine), 126.70 (2,6-C biphenyl), 126.84 (2′,6′-C biphenyl), 127.39 (2,6-C phenyl), 127.59 (4′-C biphenyl), 130.13 (3,5-C phenyl), 130.91 (3,5-C biphenyl), 131.24 (3′,5′-C biphenyl), 133.11 (1-C phenyl), 134.53 (1-C biphenyl), 135.94 (1′-C biphenyl), 139.18 (4-C biphenyl), 140.89 (4-C phenyl), 172.22 (C=O).

##### (*E*)-1-(2-([1,1′-Biphenyl]-4-yl)thiomorpholino)-3-phenylprop-2-en-1-one (**8**)

Yield: 34%, off-white solid. M.p.: 186.6–187.4 °C. ^1^H NMR (400 MHz, CDCl_3_) δ (ppm): 1.26 (s, 1H, 6-CH_2_ thiomorpholine), 2.14 (d, *J* = 20.7 Hz, 1H, 6-CH_2_ thiomorpholine), 2.68–2.73 (m, 1H, 5-CH_2_ thiomorpholine), 2.96–3.07 (m, 1H, 5-CH_2_ thiomorpholine), 3.18 (s, 1H, 3-CH_2_ thiomorpholine), 3.61 (d, *J* = 56.9 Hz, 1H, 3-CH_2_ thiomorpholine), 4.04 (s, 1H, 2-CH thiomorpholine), 6.46 (d, *J* = 16.0 Hz, ½ × 1H, ½ × 3-CH propenone), 7.37 (s, 3H, phenyl), 7.42 (s, 1H, biphenyl), 7.45 (d, *J* = 4.6 Hz, 2H, biphenyl), 7.48 (s, 2H, phenyl), 7.53–7.58 (m, 6H, biphenyl), 7.71 (d, *J* = 15.3 Hz, 1H, 2-CH propenone), 7.77 (d, *J* = 16.0 Hz, 1H, ½ × 3-CH propenone). ^13^C NMR (101 MHz, CDCl_3_) δ (ppm): 29.64 (6-C thiomorpholine), 44.49 (2-C thiomorpholine), 47.92 (5-C thiomorpholine), 50.99 (3-C thiomorpholine), 116.72 (2-C propenone), 121.80 (4-C phenyl), 127.38 (2′,6′-C biphenyl), 127.85 (2,6-C phenyl), 128.30 (4′-C biphenyl), 128.67 (2,6-C biphenyl), 128.87 (3,5-C phenyl), 129.86 (4-C biphenyl), 131.95 (3,5,3′,5′-C biphenyl), 135.05 (1-C biphenyl), 138.34 (1′-C biphenyl), 139.43 (1-C phenyl), 143.65 (3-C propenone), 165.85 (C=O).

#### 3.1.10. General Synthesis of Compounds **2**, **4**, **9,** and **10**

To a dry flask, 0.8 mmol of the respective acid **f**, **e**, nicotinic acid, or 6-hydroxy-2,5,7,8-tetramethylchroman-2-carboxylic acid is dissolved in chloroform (or tetrahydrofuran for compound **10**) and a solution of 1.0 mmol of 1,1′-carbonyldiimidazole in chloroform (or tetrahydrofuran for compound **10**) is added at room temperature (or at 0 °C for compound **9**). The reaction mixture is stirred at room temperature for 30 min. Then, a solution of 1.1 mmol of **c** in chloroform (or tetrahydrofuran for compound **10**) is added and the reaction mixture is stirred at room temperature for 30 min and at reflux for 24 h. Afterwards, the solvent is evaporated, ethyl acetate is added and the organic phase is washed with water, a saturated solution of sodium bicarbonate and hydrochloric acid 1N. The desired product is isolated with flash chromatography.

##### (*E*)-1-(2-([1,1′-Biphenyl]-4-yl)thiomorpholino)-3-(3,4-dimethoxyphenyl)prop-2-en-1-one (**2**)

Yield: 46%, off-white solid. M.p.: 172.3–173.6 °C. ^1^H NMR (400 MHz, CDCl_3_) δ (ppm): 1.42 (s, 2H, 6-CH_2_ thiomorpholine), 2.67–2.73 (m, 1H, 5-CH_2_ thiomorpholine), 3.00 (t, *J* = 11.3 Hz, 1H, 5-CH_2_ thiomorpholine), 3.16 (s, 1H, 3-CH_2_ thiomorpholine), 3.59 (d, *J* = 59.2 Hz, 1H, 3-CH_2_ thiomorpholine), 3.90 (s, 6H, 3,4-OCH_3_), 4.02 (s, 1H, 2-CH thiomorpholine), 6.70 (s, 1H, 3-CH propenone), 6.85 (d, *J* = 8.1 Hz, 1H, phenyl), 7.06 (d, *J* = 35.6 Hz, 2H, phenyl), 7.44 (dd, *J* = 14.0, 8.3 Hz, 4H, biphenyl), 7.55 (dt, *J* = 8.2, 4.3 Hz, 5H, biphenyl), 7.66 (d, *J* = 15.2 Hz, 1H, 2-CH propenone). ^13^C NMR (101 MHz, CDCl_3_) δ (ppm): 26.92 (3-OCH_3_), 29.62 (6-C thiomorpholine), 44.54 (2-C thiomorpholine), 50.89 (5-C thiomorpholine), 54.47 (3-C thiomorpholine), 56.01 (4-OCH_3_), 110.07 (2-C phenyl), 111.12 (5-C phenyl), 114.39 (2-C propenone), 121.92 (6-C phenyl), 127.34 (2′,6′-C biphenyl), 128.04 (1-C phenyl), 128.32 (4′-C biphenyl), 128.65 (2,6-C biphenyl), 131.00 (4-C biphenyl), 131.26 (1-C biphenyl), 131.95 (3,5,3′,5′-C biphenyl), 138.43 (1′-C biphenyl), 143.69 (3-C propenone), 149.15 (4-C phenyl), 150.75 (3-C phenyl), 166.04 (C=O).

##### (2-([1.1′-Biphenyl-4-yl]thiomorpholino)(3,4,5-trimethoxyphenyl)methanone (**4**)

Yield: 46%, off-white solid. M.p.: 148.4–149.6 °C. ^1^H NMR (400 MHz, CDCl_3_) δ (ppm): 1.24–1.34 (m, 1H, 6-CH_2_ thiomorpholine), 1.79 (s, 1H, 6-CH_2_ thiomorpholine), 2.66 (s, 1H, 5-CH_2_ thiomorpholine), 2.97 (s, 1H, 5-CH_2_ thiomorpholine), 3.33 (s, 1H, 3-CH_2_ thiomorpholine), 3.87 (dd, *J* = 6.8, 3.5 Hz, 9H, 3,4,5-OCH_3_), 3.93 (dd, *J* = 13.0, 6.3 Hz, 1H, 3-CH_2_ thiomorpholine), 4.11 (d, *J* = 52.3 Hz, 1H, 2-H thiomorpholine), 6.63 (d, *J* = 5.8 Hz, 2H, 2,6-H phenyl), 7.19–7.25 (m, 1H, ½ × 2′,6′-H phenyl), 7.30–7.46 (m, *J* = 37.2, 11.4 Hz, 4H, [½ × 2′,6′-H], 2,6-H, 4′-H biphenyl), 7.51 (s, *J* = 10.9 Hz, 2H, 3′,5′-H biphenyl), 7.55 (d, *J* = 8.4 Hz, 2H, 3,5-H biphenyl). ^13^C NMR (101 MHz, CDCl_3_) δ (ppm): 29.70 (6-C thiomorpholine), 56.24 (2-C thiomorpholine), 56.33 (3,5-OCH_3_), 58.44 (5-C thiomorpholine), 60.87 (3-C thiomorpholine), 60.97 (4-OCH_3_), 104.11 (2,6-C phenyl), 121.82 (1-C phenyl), 127.37 (2′,6′-C biphenyl), 128.21 (4′-C biphenyl), 128.64 (2,6-C biphenyl), 130.96 (4-C biphenyl), 131.27 (1-C biphenyl), 131.95 (3,5,3′,5′-C biphenyl), 139.32 (4-C phenyl), 139.92 (1′-C biphenyl), 153.47 (3,5-C phenyl), 170.69 (C=O).

##### (2-([1,1′-Biphenyl-4-yl]thiomorpholine)(pyridine-3-yl)methanone (**9**)

Yield: 42%, off-white solid. M.p.: 107.1–108.8 °C. ^1^H NMR (400 MHz, CDCl_3_) δ (ppm): 2.60 (s, 1H, 6-CH_2_ thiomorpholine), 2.72 (d, *J* = 14.1 Hz, 1H, 6-CH_2_ thiomorpholine), 2.91 (s, 1H, 5-CH_2_ thiomorpholine), 3.01–3.10 (m, 1H, 5-CH_2_ thiomorpholine), 3.29 (s, 1H, 2-CH thiomorpholine), 3.41–3.56 (m, 2H, 3-CH_2_ thiomorpholine), 7.22 (dd, *J* = 16.9, 8.6 Hz, 3H, biphenyl), 7.41 (dd, *J* = 9.7, 3.3 Hz, 3H, biphenyl), 7.53 (t, *J* = 7.7 Hz, 4H, biphenyl and 5-C pyridine), 7.75 (s, 1H, 4-C pyridine), 8.69 (s, 2H, 2,6-C pyridine). ^13^C NMR (101 MHz, CDCl3) δ (ppm): 14.20 (6-C thiomorpholine), 20.74 (2-C thiomorpholine), 29.69 (5-C thiomorpholine), 60.40 (3-C thiomorpholine), 123.71 (3-C pyridine, 127.38 (1-C biphenyl), 128.18 (5-C pyridine), 128.64 (2′,6′-C biphenyl), 130.96 (4′-C biphenyl), 131.27 (2,6-C biphenyl), 131.36 (4-C biphenyl), 131.95 (3,5,3′,5′-C biphenyl), 135.11 (1′-C biphenyl), 147.53 (4-C pyridine), 150.85 (6-C pyridine), 168.20 (2-C pyridine), 174.35 (C=O).

##### (2-([1,1′-Biphenyl-4-yl]thiomorpholino)(6-hydroxy-2,5,7,8-tetramethylchroman-2-yl)methanone (**10**)

Yield: 41%, off-white solid. M.p.: 109.0–109.6 C°. ^1^H NMR (400 MHz, CDCl_3_) δ (ppm): 1.61 (d, *J* = 3.8 Hz, 3H, 2′’-CH_3_), 1.71 (s, 1H, 6-CH_2_ thiomorpholine), 1.91–1.98 (m, 2H, 3′′-CH_2_), 2.08 (s, 3H, 8′′-CH_3_), 2.17 (d, *J* = 5.0 Hz, 6H, 5′,7′-CH_3_), 2.54–2.61 (m, *J* = 15.7 Hz, 2H, 4′′-CH_2_), 2.62–2.71 (m, 2H, 5-CH_2_ thiomorpholine), 2.99 (s, 1H, 6-CH_2_ thiomorpholine), 3.34 (s, 1H, 3-CH_2_ thiomorpholine), 3.65–3.77 (m, 1H, 3-CH_2_ thiomorpholine), 3.99 (s, 1H, 2-CH thiomorpholine), 7.25 (s, 1H, thiomorpholine), 7.27 (s, 1H, thiomorpholine), 7.29–7.34 (m, 1H, thiomorpholine), 7.36–7.46 (m, 2H, thiomorpholine), 7.55 (d, *J* = 7.9 Hz, 3H, thiomorpholine), 7.64 (s, 1H, thiomorpholine). ^13^C NMR (101 MHz, CDCl_3_) δ (ppm): 11.29 (8-CH_3_), 12.21 (5,7-CH_3_), 21.09 (2-CH_3_), 24.40 (6-C thiomorpholine), 25.51 (2-C thiomorpholine), 25.63 (3-C chromane), 29.71 (4-C chromane), 31.55 (5-C thiomorpholine), 51.68 (3-C thiomorpholine), 79.29 (2-C chromane), 117.99 (4a-C chromane), 119.08 (5-C chromane), 121.39 (7-C chromane), 122.48 (8-C chromane), 127.08 (1-C biphenyl), 128.13 (4′-C biphenyl), 128.64 (2′,6′-C biphenyl), 131.23 (3,5,3′,5′-C biphenyl), 131.92 (4-C biphenyl), 132.45 (2,6-C biphenyl), 139.38 (1′-C biphenyl), 144.64 (6-C chromane), 145.60 (8a-C chromane), 172.60 (C=O).

#### 3.1.11. Synthesis of Compound (*E*)-1-(2-([1.1′-Biphenyl]-4-yl)thiomorpholino)-3-(3,5-ditertbutyl-4-hydroxyphenyl)prop-2-en-1-one (**6**)

To a dry flask, 0.59 mmol of **c** is dissolved in dichloromethane. Then, 0.32 mmol of (*E*)-3-(3,5-ditertbutyl-4-hydroxyphenyl)acrylic acid and 0.62 mmol of 4-dimethylaminopyridine are added at 0 °C. Then, 0.62 mmol of N,N’-dicyclohexylcarbodiimide is added gradually and the reaction mixture is stirred at 0 °C for 30 min and at room temperature for 16 h. Afterwards, it is diluted with dichloromethane and the precipitate is filtered. The filtrate is washed with water and saturated solution of sodium bicarbonate, dried over sodium sulfate, and evaporated. The desired product is isolated with flash chromatography. Yield: 83%, orange solid. M.p.: 112.2–115.9 °C. ^1^H NMR (400 MHz, CDCl_3_) δ (ppm): 1.38 (s, 9H, 3′′ ή 5′′-C(CH_3_)_3_), 1.49 (s, 9H, 3′′ ή 5′′-C(CH_3)3_), 1.56–1.64 (m, 1H, 6-CH_2_ thiomorpholine), 1.71 (dd, *J* = 9.5, 3.9 Hz, 1H, 6-CH_2_ thiomorpholine), 1.90 (dd, *J* = 26.1, 11.7 Hz, 2H, 5-CH_2_ thiomorpholine), 2.70 (d, *J* = 11.3 Hz, 1H, 3-CH_2_ thiomorpholine), 2.98 (d, *J* = 15.7 Hz, 1H, 3-CH_2_ thiomorpholine), 3.44 (t, *J* = 10.2 Hz, 1H, 2-CH thiomorpholine), 6.48 (dd, *J* = 54.7, 15.9 Hz, 1H, 3-CH propenone), 7.28 (d, *J* = 6.4 Hz, 1H, biphenyl), 7.33–7.40 (m, 2H, biphenyl), 7.41–7.49 (m, 3H, biphenyl), 7.55 (dd, *J* = 8.3, 5.3 Hz, 3H, biphenyl), 7.71 (dd, *J* = 20.7, 14.1 Hz, 2H, phenyl), 7.82 (dd, *J* = 15.3, 8.8 Hz, 1H, 2-CH propenone). ^13^C NMR (101 MHz, CDCl_3_) δ (ppm): 29.70 (6-C thiomorpholine), 30.16 (3,5-C(CH_3_)_3_), 31.44 (3,5-C(CH_3_)_3_), 32.86 (2-C thiomorpholine), 34.33 (5-C thiomorpholine), 35.60 (3-C thiomorpholine), 113.20 (2-C propenone), 125.13 (2′,6′-C biphenyl), 125.95 (1-C phenyl), 126.36 (4′-C biphenyl), 126.96 (1-C biphenyl), 131.02 (2,6-C biphenyl), 131.25 (3,5,3′,5′-C biphenyl), 132.49 (2,6-C phenyl), 136.34 (3,5-C phenyl), 136.63 (1′-C biphenyl), 136.65 (4-C biphenyl), 145.00 (3-C propenone), 155.79 (4-C phenyl), 166.43 (C=O).

#### 3.1.12. Synthesis of Compound 2-([1,1′-Biphenyl]-4-yl)-3,4-dihydro-2H-benzo[b][1,4]thiazine (**11**)

To a dry flask, 3.5 mmol of **d** is dissolved in dry tetrahydrofuran, under inert atmosphere, and 14 mmol of borane in tetrahydrofuran (1M) complex is added dropwise. The reaction is stirred under these conditions for 4 days, diluted with cold water under cooling (0 °C) and evaporated. Afterwards, the aqueous phase is extracted with dichloromethane, dried over sodium sulfate, and evaporated. The desired product is purified via flash chromatography. Yield: 80%, yellow solid. M.p.: 97.2–99.0 °C. ^1^H NMR (400 MHz, CDCl_3_) δ (ppm): 3.63 (ddd, *J* = 12.1, 9.0, 5.7 Hz, 1H, 3-CH_2_ benzothiazine), 3.79 (dd, *J* = 12.0, 3.0 Hz, 1H, 3-CH_2_ benzothiazine), 4.38 (ddd, *J* = 16.9, 8.7, 3.0 Hz, 1H, 2-CH benzothiazine), 6.58 (dd, *J* = 8.0, 1.2 Hz, 1H, 5-CH benzothiazine), 6.69 (tdd, *J* = 7.6, 3.9, 1.2 Hz, 1H, 6-CH benzothiazine), 6.93–6.98 (m, 1H, 7-CH benzothiazine), 7.06 (dd, *J* = 7.8, 1.4 Hz, 1H, 8-CH benzothiazine), 7.28 (dd, *J* = 6.2, 2.3 Hz, 2H, 2′,6′-CH biphenyl), 7.36–7.39 (m, 1H, 3′,5′-CH biphenyl), 7.45 (dd, *J* = 7.0, 1.6 Hz, 1H, 3′,5′-CH biphenyl), 7.52–7.58 (m, 4H, 2,3,5,6-CH biphenyl), 7.68 (d, *J* = 1.8 Hz, 1H, 4′-CH biphenyl). ^13^C NMR (101 MHz, CDCl_3_) δ (ppm): 42.81 (2-C benzothiazine), 48.79 (3-C benzothiazine), 115.46 (7-C benzothiazine), 118.53 (8a-C benzothiazine), 125.67 (6-C benzothiazine), 127.21 (4′-C biphenyl), 128.56 (5-C benzothiazine), 128.67 (4-C biphenyl), 131.04 (2,6,2′,6′-C biphenyl), 131.26 (3,5,3′,5′-C biphenyl), 131.95 (1-C biphenyl), 132.66 (1′-C biphenyl), 139.43 (8-C benzothiazine), 141.07 (4a-C benzothiazine).

#### 3.1.13. General Synthesis of Compounds **12**, **14**–**17**

To a dry flask, 1.00 mmol of **11** is dissolved in chloroform and 1.5 mmol of triethylamine is added. Then, 1.2–2.0 mmol of each chloride **o**, **p**, **l**, **n,** or **m** is added dropwise, dissolved in chloroform. The reaction mixture is stirred at room temperature or reflux for 1–24 h. Afterwards, the reaction is diluted with chloroform and the organic phase is washed with water, dried over sodium sulfate and evaporated. The desired product is isolated with flash chromatography.

##### (*E*)-1-(2-([1,1′-Biphenyl]-4-yl)-2,3-dihydro-4H-benzo[b][1,4]thiazin-4-yl)-3-(3,4-dimethoxyphenyl)prop-2-en-1-one (**12**)

Yield: 74%, off-white solid. M.p.: 90.8–91.9 °C. ^1^H NMR (400 MHz, CDCl_3_) δ (ppm): 3.45 (s, 1H, 3-CH_2_ benzothiazine), 3.86 (s, 3H, 3-OCH_3_), 3.90 (s, 3H, 4-OCH_3_), 4.77 (ddd, *J* = 14.5, 10.2, 4.4 Hz, 1H, 3-CH_2_ benzothiazine), 5.04 (s, 1H, 2-CH benzothiazine), 6.70 (dd, *J* = 15.5, 2.6 Hz, 1H, 3-CH propenone), 6.84 (d, *J* = 8.4 Hz, 1H, 5-CH benzothiazine), 6.93 (d, *J* = 1.7 Hz, 1H, 6-CH benzothiazine), 7.06 (dd, *J* = 8.4, 1.8 Hz, 1H, 7-CH benzothiazine), 7.15 (ddd, *J* = 5.3, 4.7, 1.5 Hz, 1H, 8-CH benzothiazine), 7.18–7.24 (m, 3H, phenyl), 7.25–7.29 (m, 3H, biphenyl), 7.35 (dd, *J* = 7.8, 1.4 Hz, 1H, biphenyl), 7.44 (dd, *J* = 8.0, 1.7 Hz, 1H, biphenyl), 7.54 (dd, *J* = 6.9, 4.8 Hz, 3H, biphenyl), 7.71 (d, *J* = 15.4 Hz, 1H, 2-CH propenone), 7.77 (d, *J* = 1.6 Hz, 1H, 4′-CH biphanyl). ^13^C NMR (101 MHz, CDCl_3_) δ (ppm): 26.92 (3,4-OCH_3_), 47.07 (2-C benzothiazine), 55.97 (3-C benzothiazine), 110.28 (2-C phenyl), 111.11 (5-C phenyl), 116.22 (2-C propenone), 122.10 (7-C benzothiazine), 122.70 (8a-C benzothiazine), 124.52 (6-C benzothiazine), 126.67 (4′-C benzothiazine), 127.18 (6-C phenyl), 128.68 (5-C benzothiazine), 129.46 (1-C phenyl), 131.02 (2,6,2′,6′-C biphenyl), 131.24 (3,5,3′,5′-C biphenyl), 132.71 (4-C biphenyl), 137.03 (8-C benzothiazine), 139.31 (1-C biphenyl), 139.90 (4a-C benzothiazine), 141.31 (1′-C biphenyl), 143.32 (3-C propenone), 149.14 (4-C phenyl), 150.88 (3-C phenyl), 165.38 (C=O).

##### (2-([1,1′-Biphenyl]-4-yl)-2,3-dihydro-4H-benzo[b][1,4]thiazin-4-yl)(3,4,5-trimethoxyphenyl)methanone (**14**)

Yield: 78%, off-white solid. M.p.: 106.2–107.5 °C. ^1^H NMR (400 MHz, CDCl_3_) δ (ppm): 3.58 (s, 1H, 3-CH_2_ benzothiazine), 3.64 (s, 6H, 3,5-OCH_3_), 3.81 (s, 3H, 4-OCH_3_), 4.83 (dd, *J* = 9.8, 4.8 Hz, 1H, 3-CH_2_ benzothiazine), 4.95 (dd, *J* = 12.4, 4.1 Hz, 1H, 2-CH benzothiazine), 6.56 (s, 2H, 2,6-CH phenyl), 6.65 (t, *J* = 8.5 Hz, 1H, 5-CH benzothiazine), 6.84 (t, *J* = 7.7 Hz, 1H, 6-CH benzothiazine), 7.04 (dd, *J* = 11.1, 4.1 Hz, 1H, 7-CH benzothiazine), 7.24 (d, *J* = 1.7 Hz, 1H, 8-CH benzothiazine), 7.27–7.32 (m, 2H, biphenyl), 7.44 (dt, *J* = 10.6, 5.1 Hz, 2H, biphenyl), 7.53 (d, *J* = 8.0 Hz, 4H, biphenyl), 7.78 (d, *J* = 1.2 Hz, 1H, 4′-CH biphenyl). ^13^C NMR (101 MHz, CDCl_3_) δ (ppm): 47.92 (2-C benzothiazine), 48.86 (3-C benzothiazine), 56.03 (3,5-OCH_3_), 60.93 (4-OCH_3_), 106.64 (2,6-C phenyl), 122.19 (7-C benzothiazine), 122.73 (8a-C benzothiazine), 124.86 (1-C phenyl), 126.04 (6-C benzothiazine), 126.70 (4′-C biphenyl), 128.39 (5-C benzothiazine), 131.01 (2,6,2′,6′-C biphenyl), 131.27 (3,5,3′,5′-C biphenyl), 132.76 (4-C biphenyl), 138.87 (4-C phenyl), 139.23 (8-C benzothiazine), 139.91 (1-C biphenyl), 140.08 (1′-C biphenyl), 141.37 (4a-C benzothiazine), 152.68 (3,5-C phenyl), 168.66 (C=O).

##### (2-([1,1′-Biphenyl]-4-yl)-2,3-dihydro-4H-benzo[b][1,4]thiazin-4-yl)(3,5-ditertbutyl-4-hydroxyphenyl)methanone (**15**)

Yield: 51%, orange solid. M.p.: 94.6–95.6 °C. ^1^H NMR (400 MHz, CDCl_3_) δ (ppm): 1.29 (s, 18H, 3,5-C(CH_3_)_3_), 3.57–3.64 (m, 1H, 3-CH_2_ benzothiazine), 4.84 (dd, *J* = 9.8, 4.8 Hz, 1H, 3-CH_2_ benzothiazine), 4.99 (d, *J* = 9.1 Hz, 1H, 2-CH benzothiazine), 5.44 (s, 1H, -OH), 6.58 (d, *J* = 8.1 Hz, 1H, 5-CH benzothiazine), 6.81–6.85 (m, 1H, 6-CH benzothiazine), 7.02–7.07 (m, 1H, 7-CH benzothiazine), 7.21 (s, 2H, 2,6-CH phenyl), 7.26–7.34 (m, 4H, 8-CH benzothiazine, biphenyl), 7.45 (ddd, *J* = 8.6, 6.4, 1.9 Hz, 2H, biphenyl), 7.53–7.57 (m, 3H, biphenyl), 7.80 (d, *J* = 1.7 Hz, 1H, 4′-CH biphenyl). ^13^C NMR (101 MHz, CDCl_3_) δ (ppm): 30.02 (3,5-C(CH_3_)_3_), 34.21 (3,5-C(CH_3_)_3_), 48.05 (2-C benzothiazine), 48.62 (3-C benzothiazine), 122.12 (7-C benzothiazine), 122.68 (8a-C benzothiazine), 124.40 (1-C phenyl), 125.56 (6-C benzothiazine), 126.61 (4′-C biphenyl), 127.16 (2,6-C phenyl), 128.47 (5-C benzothiazine), 131.05 (2,6,2′,6′-C biphenyl), 131.24 (3,5,3′,5′-C biphenyl), 132.76 (4-C biphenyl), 135.16 (3,5-C phenyl), 139.33 (8-C benzothiazine), 139.59 (1-C biphenyl), 140.24 (1′-C biphenyl), 141.22 (4a-C benzothiazine), 156.12 (4-C phenyl), 169.60 (C=O).

##### (*E*)-1-(2-([1,1′-Biphenyl]-4-yl)-2,3-dihydro-4H-benzo[b][1,4]thiazin-4-yl)-3-(3,5-ditertbutyl-4-hydroxyphenyl)prop-2-en-1-one (**16**)

Yield: 81%, orange solid. M.p.: 115.8–117.6 °C. 1H NMR (400 MHz, CDCl3) δ (ppm): 1.40 (s, 18H, 3,5-C(CH_3_)_3_), 3.42 (s, 1H, 3-CH_2_ benzothiazine), 4.75 (ddd, *J* = 14.5, 10.3, 4.3 Hz, 1H, 3-CH_2_ benzothiazine), 5.05 (s, 1H, 2-CH benzothiazine), 6.67 (d, *J* = 15.4 Hz, 1H, 3-CH propenone), 7.10 (ddd, *J* = 7.7, 3.7, 1.3 Hz, 1H, 5-CH benzothiazine), 7.18 (ddd, *J* = 6.2, 4.3, 3.1 Hz, 2H, 6,7-CH benzothiazine), 7.24 (s, 2H, biphenyl), 7.25 (s, 2H, 2,6-CH phenyl), 7.29–7.34 (m, 1H, 8-CH benzothiazine), 7.40–7.45 (m, 2H, biphenyl), 7.48–7.55 (m, 4H, biphenyl), 7.69 (d, *J* = 15.4 Hz, 1H, 2-CH propanone), 7.75 (d, *J* = 1.5 Hz, 1H, 4′-CH biphenyl). ^13^C NMR (101 MHz, CDCl_3_) δ (ppm): 30.13 (3,5-C(CH_3_)_3_), 34.31 (3,5-C(CH_3_)_3_), 47.03 (2-C benzothiazine), 47.53 (3-C benzothiazine), 115.24 (2-C propenone), 122.11 (7-C benzothiazine), 122.68 (8a-C benzothiazine), 124.29 (1-C phenyl), 125.37 (2,6-C phenyl), 126.40 (6-C benzothiazine), 127.20 (4′-C biphenyl), 128.70 (5-C benzothiazine), 131.04 (2,6,2′,6′-C biphenyl), 131.24 (3,5,3′,5′-C biphenyl), 132.74 (4-C biphenyl), 136.32 (3,5-C phenyl), 137.07 (8-C benzothiazine), 139.34 (1-C biphenyl), 139.96 (1′-C biphenyl), 141.28 (4a-C benzothiazine), 144.33 (3-C propenone), 155.87 (4-C phenyl), 165.68 (C=O).

##### 1-(2-([1,1′-Biphenyl]-4-yl)-2,3-dihydro-4H-benzo[b][1,4]thiazin-4-yl)-2-(4-isobutylphenyl)propan-1-one (**17**)

Yield: 51%, off-white solid. M.p.: 127.2–129.6 °C. ^1^H NMR (400 MHz, CDCl_3_) δ (ppm): 0.85–0.88 (m, 6H, -CH(CH_3_)_2_), 1.51 (d, *J* = 6.8 Hz, 3H, -CH(CH_3_)), 1.80 (dp, *J* = 13.5, 6.8 Hz, 1H, -CH(CH_3_)_2_), 2.39 (dd, *J* = 7.1, 1.9 Hz, 2H, -CHCH_2_-), 2.92 (d, *J* = 27.9 Hz, 1H, -CH(CH_3_)), 4.27 (s, 1H, 3-CH_2_ benzothiazine), 4.45–4.54 (m, 1H, 3-CH_2_ benzothiazine), 4.93 (s, 1H, 2-CH benzothiazine), 6.80 (s, 2H, phenyl), 6.94 (d, *J* = 7.6 Hz, 2H, phenyl), 7.09–7.20 (m, 4H, 5,6,7,8-CH benzothiazine), 7.21–7.25 (m, 2H, biphenyl), 7.27 (s, 1H, biphenyl), 7.36 (dd, *J* = 8.0, 1.7 Hz, 1H, biphenyl), 7.41–7.45 (m, 1H, biphenyl), 7.47–7.57 (m, 3H, biphenyl), 7.69 (d, *J* = 1.7 Hz, 1H, 4′-CH biphenyl). ^13^C NMR (101 MHz, CDCl_3_) δ (ppm): 22.40 (-CH(CH_3_)), 22.45 (-CH(CH_3_)_2_), 30.36 (-CH(CH_3_)_2_), 42.58 (-CH(CH_3_)), 45.09 (-CH_2_CH(CH_3_)_2_), 46.04 (2-C benzothiazine), 48.34 (3-C benzothiazine), 122.23 (7-C benzothiazine), 122.71 (8a-C benzothiazine), 124.65 (6-C benzothiazine), 126.76 (4′-C biphenyl), 126.97 (2,6-C phenyl), 127.29 (5-C benzothiazine), 128.78 (4-C biphenyl), 129.40 (3,5-C phenyl), 131.12 (2,6,2′,6′-C phenyl), 131.36 (3,5,3′,5′-C phenyl), 132.04 (1-C biphenyl), 132.80 (1′-C biphenyl), 137.55 (1-C phenyl), 139.45 (8-C benzothiazine), 140.30 (4-C phenyl), 141.26 (4a-C benzothiazine), 174.39 (C=O).

#### 3.1.14. Synthesis of Compound (2-([1,1′-Biphenyl]-4-yl)-2,3-dihydro-4H-benzo[b][1,4]thiazin-4-yl)(3,4,5-trihydroxyphenyl)methanone (**13**)

To a dry flask, 1.0 mmol of **11** is dissolved in chloroform and 1.5 mmol of triethylamine is added. Then, 2.0 mmol of **i** is added dropwise and dissolved in chloroform. The reaction mixture is stirred at room temperature for 3 h. Afterwards, it is diluted with chloroform and the organic phase is washed with water, dried over sodium sulfate, and evaporated. The benzoyl group is deprotected by treatment with potassium carbonate 1N in methanol/water at a ratio of 9:1. The reaction mixture is stirred at room temperature for 2 h. Afterwards, the methanol is evaporated at 40 °C and the residue is extracted with ethyl acetate. The organic phase is dried over magnesium sulfate and evaporated. Total yield: 20%, brown solid. M.p.: 97.3–98.4 °C. ^1^H NMR (400 MHz, CDCl_3_) δ (ppm): 3.69 (s, 1H, 3-CH_2_ benzothiazine), 4.77 (s, 1H, 3-CH_2_ benzothiazine), 4.96 (dd, *J* = 24.5, 13.2 Hz, 1H, 2-CH benzothiazine), 6.47 (s, 2H, 2,6-CH phenyl), 6.53 (s, 1H, 5-CH benzothiazine), 6.76 (s, 1H, 6-CH benzothiazine), 6.86 (s, 1H, 7-CH benzothiazine), 7.07 (d, *J* = 7.7 Hz, 1H, 8-CH benzothiazine), 7.35–7.43 (m, 3H, biphenyl), 7.54 (t, *J* = 7.5 Hz, 4H, biphenyl), 7.65 (s, 1H, biphenyl), 7.74 (s, 1H, 4′-CH biphenyl). ^13^C NMR (101 MHz, CDCl_3_) δ (ppm): 47.50 (2-C benzothiazine), 60.58 (3-C benzothiazine), 109.20 (2,6-C phenyl), 122.31 (7-C benzothiazine), 122.86 (8a-C benzothiazine), 124.89 (1-C phenyl), 126.44 (6-C benzothiazine), 127.05 (4′-C biphenyl), 128.83 (5-C benzothiazine), 131.17 (2,6,2′,6′-C biphenyl), 131.41 (3,5,3′,5′-C biphenyl), 132.09 (8-C benzothiazine), 132.86 (4-C biphenyl), 139.45 (1-C biphenyl), 140.03 (1′-C biphenyl), 141.52 (4a-C benzothiazine), 144.01 (3,4,5-C phenyl), 169.26 (C=O).

### 3.2. Pharmacological Evaluation

#### 3.2.1. Lipoxygenase (LOX) Inhibition

Lipoxygenase (LOX) inhibitory activity was determined using soybean lipoxygenase (250 U/mL) and sodium linoleate (100 μM) as substrate, in Tris–HCl buffer pH 9.0. The test compounds dissolved in methanol were added and the reaction was monitored for 5 min at 28 °C, recording absorbance at 234 nm. Each concentration was evaluated twice and results were expressed as IC_50_ (μM) after incubation for 5 min [44].

#### 3.2.2. Edema Reduction

For the in vivo anti-inflammatory activity, C57BL/6 mice were injected with 0.025 mL carrageenan (2% *w*/*v* solution in saline) *i.d.* into the right hind paw, the left paw serving as control. The test compounds dissolved in saline (300 μmol/kg) were given *i.p.* right after the carrageenan injection, and 3.5 h later the produced edema was estimated as paw weight increase. Results are expressed as percentage of reduction in paw-edema and are the mean of 6 animals (per compound) [45].

#### 3.2.3. Radical Scavenging of DPPH

Compounds, dissolved in absolute methanol, at concentrations of 25–400 μM, were added to an equal volume of a methanolic solution of DPPH (final concentration 200 μM) at room temperature (22 ± 2 °C). Absorbance (517 nm) was recorded at different time intervals for up to 1 h and results are expressed as IC_50_ (μM) for DPPH interaction after incubation for 30 min [32].

#### 3.2.4. Inhibition of Lipid Peroxidation

The incubation mixture contained heat-inactivated (90 °C for 90 s) liver microsomal fraction from untreated C57BL/6 mice (corresponding to 2.5 mg of hepatic protein per milliliter or 4 mM fatty acid residues), ascorbic acid (0.2 mM) in Tris–HCl/KCl buffer (50 mM/150 mM, pH 7.4), and the studied compounds dissolved in dimethyl sulfoxide (in different concentrations). The reaction was initiated by the addition of a freshly prepared FeSO_4_ solution (10 μM) and the mixture was incubated at 37 °C for 45 min. Aliquots were taken at various time intervals and lipid peroxidation was assessed by spectrophotometric (535 against 600 nm) determination of the produced 2-thiobarbituric acid reactive material [32]. Each concentration was evaluated twice and results were expressed as IC_50_ (μM) after incubation for 45min.

#### 3.2.5. Hypolipidaemic Activity

An aqueous solution of Triton WR 1339 was given *i.p.* to mice (400 mg/kg of body weight) and one hour later the test compounds (84 μmol/kg of body weight), dissolved in saline or saline only, were administered *i.p*. After 24 h, blood was taken from the aorta/heart and used for the determination of plasma total cholesterol (TC), triglyceride (TG) levels, and high-density lipoprotein (HDL) levels, using commercially available kits (Biosis Cholesterol Enzymatic/PAP, Biosis Triglycerides GPO/PAP, and Biosis HDL Phosphovolframic, Biosis, Athens Greece). Levels of plasma lipids were determined in duplicate while values presented are the mean from 8 animals (per compound). The levels of low-density lipoprotein (LDL) are calculated according to the equation: LDL (mg/dL) = 3/4 * (TC-HDL) [24].

#### 3.2.6. Acetylcholinesterase (AChE) Inhibition

A reaction mixture is formed containing a final concentration of 300 μM for each tested compound in methanol, a solution of the enzyme acetylcholinesterase from eel (2333 U/mg) (Sigma), an aqueous solution of acetylthiocholine as substrate (0.47 mM), and Ellman’s reagent [(5,5′-bisthio-bis-(2-nitrobenzoic acid))] (0.31 mM) in phosphate buffer with pH = 7. The incubation mixture is monitored for 7 min at 37 °C. Each result expresses the mean absorbance (412 nm) of at least 2 tests per sample.

#### 3.2.7. Iron Chelation (Ferrozine)

A reaction mixture is formed containing the tested compound at a concentration of 100μM in ammonium acetate 5% *w*/*v*, with pH = 6.9 and a Fe^2+^ solution at a concentration of 20 μM. The reaction was initiated with the addition of a 100 μM aqueous ferrozine solution, which chelates iron ions, resulting in an increased maximum absorbance at 562 nm. After incubation at 37 °C for 10 min, the mixture’s absorbance is measured at 562/700 nm. Each value is expressed as the mean of 3 tests per sample [46].

### 3.3. Physicochemical Properties and logBB Calculation

The physicochemical properties Molecular weight, ClogP, and TPSA of the new compounds were calculated, using the software ChemBioDrawUltra12, whereas their logBB scores were calculated via the equation: logBB = 0.5159 × ClogP − 0.0277 × TPSA − 0.3462 ≥ 0.3 (CNS+) [47].

## 4. Conclusions

The present study successfully demonstrates the design, synthesis, and biological evaluation of a new class of multitarget (benzo)thiazine derivatives that combine antioxidant, anti-inflammatory, antidyslipidaemic, as well as potential neuroprotective properties within a single molecular scaffold. This rationally designed series was developed by integrating structural motifs known to modulate key biological pathways implicated in the pathogenesis of neurodegenerative and cardiovascular diseases—namely oxidative stress, lipid dysregulation, and chronic inflammation.

Extensive in vitro and in vivo evaluations confirmed that many of the synthesized compounds display a robust multifunctional profile. Several derivatives inhibited lipoxygenase with IC_50_ values below 100 μM, reduced carrageenan-induced edema by up to 60%, and showed pronounced activity in assays assessing radical scavenging, lipid peroxidation, and iron chelation. Importantly, in vivo experiments in hyperlipidemic mice revealed a remarkable capacity of these molecules to lower total cholesterol and triglyceride levels, significantly improve the HDL/LDL ratio (antiatherogenic index up to 700% increase), and enhance systemic antioxidant capacity by more than 100%, with some compounds achieving an increase of up to 800%. In addition, moderate inhibition of acetylcholinesterase activity was observed, suggesting that these compounds may confer neuroprotective benefits through multiple converging mechanisms.

The balanced multitarget activity of the most potent derivatives—particularly compounds **3**, **13**, **15**, and **16**—highlights their potential as lead candidates for the development of therapeutics aimed at complex, multifactorial disorders. These results not only validate the (benzo)thiazine scaffold as a versatile pharmacophore but also emphasize the value of integrating lipid-regulatory, anti-inflammatory, and antioxidant features into a single molecular framework to achieve synergistic biological outcomes. This further underlines the importance of using complex systems, such as in vivo assays, in order to expose the privileged multifunctional nature of such compounds.

It is noteworthy that the favorable physicochemical properties and predicted high likelihood of blood–brain barrier permeability, reinforce the relevance of these molecules for central nervous system applications. The multitarget-directed design approach presented here aligns with current trends in medicinal chemistry that seek to address the multifaceted nature of chronic degenerative diseases through single agents capable of modulating multiple pathogenic pathways simultaneously.

Collectively, our findings establish multitarget (benzo)thiazine derivatives as a promising new chemical class with strong potential for therapeutic development against neurodegenerative and cardiometabolic disorders.

## Figures and Tables

**Table 1:** Structures of novel compounds **1**–**17**.

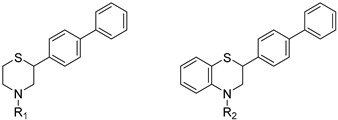			
**Compound**	R_1_ **=**	**Compound**	R_1_ **=**
**1**	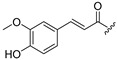	**10**	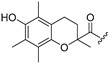
**2**	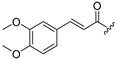		R_2_ **=**
**3**	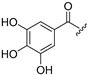	**11**	H
**4**	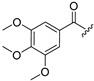	**12**	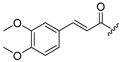
**5**	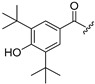	**13**	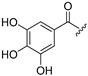
**6**	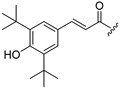	**14**	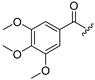
**7**	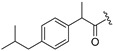	**15**	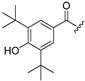
**8**	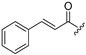	**16**	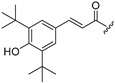
**9**	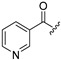	**17**	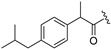

**Table 2:** Effect of the derivatives on LOX-3, expressed as IC_50_ (μM), after 5 min of incubation.

Compound	IC_50_ Value (μM)	Compound	IC_50_ Value (μM)
**1**	68	**11**	134
**2**	>300	**12**	74
**3**	4	**13**	37
**4**	71	**14**	71
**5**	150	**15**	83
**6**	252	**16**	58
**7**	22	**17**	104
**8**	>200 ^a^	cinnamic acid	>300
**9**	192	ferulic acid	132
**10**	140	ibuprofen	>300
		naproxen	25

^a^ Insufficient solubility at higher concentrations, no significant inhibition at that concentration. IC_50_ values are the mean of *n* = 3 with SEM < 10% of the respective value.

**Table 3:** In vitro antioxidant profile of the new compounds, expressed as IC_50_ values for DPPH and lipid peroxidation (LP). Percentage (%) of Fe (II) chelation at 100 μM of compound concentration.

Compound	DPPH IC_50_ Value (μM)	LP IC_50_ Value (μM) or % Inhibition at 100 μΜ	% of Fe(II) Chelation (at 100 μM)
**1**	166	6	19
**2**	>400	>1000	18
**3**	31	8	89 (56% at 10 μM)
**4**	>400	>500	4
**5**	300	250	0
**6**	288	>500	0
**7**	>400	299	3
**8**	>400	>1000	5
**9**	>400	>1000	2
**10**	78	4	0
**11**	>400	30	10
**12**	>400	>1000	17
**13**	36	24	91 (12% at 10 μM)
**14**	>400	>1000	6
**15**	>400	>500	8
**16**	141	>1000	9
**17**	>400	>1000	29
**Trolox**	33	43%	29
**BHT**	31	0%	0
**gallic acid**	14	73%	98
**ascorbic acid**	42	30	n.t

Each value represents the mean obtained from triplicate measurements for each sample, with SEM < 10% of the respective value. n.t.: not tested.

**Table 4:** Percentage (%) of increase in total antioxidant capacity (TAC) in the plasma of hyperlipidaemic mice, after the administration of the tested compounds.

Compound	Total Antioxidant Capacity (TAC) Increase (%)	Compound	Total Antioxidant Capacity (TAC) Increase (%)
**1**	329 **	**11**	199 ***
**2**	43 *	**12**	203 ***
**3**	780 ***	**13**	60
**4**	87	**14**	144 *
**5**	0	**15**	131 ***
**6**	808 ***	**16**	262 ***
**7**	0	**17**	139 **
**8**	104 ***		
**9**	60 *		
**10**	296 *		

The tested compounds were administered i.p. at a dose of 84 μmol/kg of body weight. Each value represents the mean obtained from eight animals. Significant difference from control samples: * *p* < 0.05, ** *p* < 0.005, *** *p* < 0.0005.

**Table 5:** Percentage (%) of acetylcholinesterase inhibition by the tested compounds at a concentration of 300 μM.

Compound	AChE Inhibition (%)	Compound	AChE Inhibition (%)
**1**	22	**11**	n.a.
**2**	26	**12**	17
**3**	10	**13**	43
**4**	30	**14**	47
**5**	24	**15**	23
**6**	43	**16**	24
**7**	27	**17**	13
**8**	19	**donepezil**	100
**9**	33	**tacrine**	100
**10**	45	**galanthamine**	100

Each value represents the mean obtained from triplicate measurements for each sample. Insufficient solubility at higher concentrations. IC_50_ values are the mean of n = 3 with SEM < 8% of the respective value. n.a.: not active.

**Table 6:** Percentage (%) of increase or decrease in the levels of lipidaemic parameters measured in the plasma of hyperlipidaemic mice, after the administration of the tested compounds.

Compound	% Total Cholesterol (TC) Reduction	% Triglycerides (TG) Reduction	% HDL Increase	% LDL Decrease	% HDL/LDL Increase
**1**	40 *	83 ****	84 **	59 **	242 **^,1,2^
**2**	30 *	60 **	44 **	53 **	122 ***
**3**	51 ***	86 ****	49 ***	79 ***	682 ***
**4**	10	35 *	95 **	5	n.a.
**5**	55 *	50	n.t.	n.t.	n.t.
**6**	36 **	65 ****	23 **^,2^	49 **	43 *
**7**	27 *	70 **	n.t.	n.t.	n.t.
**8**	45 **	53 **	9	57 *	91 *
**9**	35 *	33 *^,1^	n.a.	44 *	105 *
**10**	13	52 *	32	23	77
**11**	23 *	59 **	101 *	48 ***	232 **
**12**	27 *	29	63 ****	59 ***	282 ***
**13**	47 ****	53 *	25 *	62 ****	442 **
**14**	51 ****	50 *	13	73 ****	320 ****
**15**	55 ****	35	79 **	74 ****	714 ****
**16**	45 ****	67 **	131 ****	66 ****	604 ****
**17**	45 ****	58 *	43 **	68 ****	321 ***

The tested compounds were administered i.p. at a dose of 84 μmol/kg of body weight. Each value represents the mean obtained from eight animals. Significant difference from control samples: * *p* < 0.05, ** *p* < 0.01, *** *p* < 0.001, **** *p* < 0.0001. n.t.: not tested, n.a.: not active, ^1^ increase percentage, ^2^ decrease percentage.

**Table 7:** Physicochemical properties and logBB values for the new compounds.

Compound	MW (g/mol)	ClogP	H-Bond Donors	H-Bond Acceptors	TPSA	logBB
**1**	431.55	5.13	1	3	49.77	0.9
**2**	445.58	5.60	0	4	38.77	1.5
**3**	407.48	3.77	3	2	81.00	−0.6
**4**	449.57	4.46	0	5	48.00	0.6
**5**	487.70	8.28	1	2	40.54	2.8
**6**	513.74	8.73	1	2	40.54	3.0
**7**	443.65	7.69	0	2	20.31	3.1
**8**	385.53	5.95	0	2	20.31	2.2
**9**	360.48	3.85	0	3	32.67	0.7
**10**	487.66	7.38	1	3	49.77	2.1
**11**	303.42	5.56	1	1	12.03	2.2
**12**	493.62	7.92	0	4	38.77	2.7
**13**	455.53	5.24	3	2	81.00	0.1
**14**	497.61	5.92	0	5	48.00	1.4
**15**	535.75	9.74	1	2	40.54	3.6
**16**	561.78	11.05	1	2	40.54	4.2
**17**	491.69	9.18	0	2	20.31	3.8

MW: Molecular weight; ClogP: calculated log P; TPSA: topological polar surface area.

## Data Availability

The data presented in this study is available on request from the corresponding author.

## Supplementary Materials

The following supporting information can be downloaded at: https://www.mdpi.com/article/10.3390/molecules30234542/s1, Table S1: Structures of the new compounds.

## Abbreviations

The following abbreviations are used in this manuscript:

AChE	Acetylcholinesterase
ALS	Amyotrophic lateral sclerosis
BBB	Blood–brain barrier
BHT	Butylated hydroxytoluene
CDI	N,N’-carbonyldiimidazole
CNS	Central nervous system
DCC	N/N’-dicyclohexylcarbodiimide
DPPH	2,2-diphenyl-1-picrylhydrazyl
HDL	High-density lipoprotein
LDL	Low-density lipoprotein
LOX	Lipoxygenase
LP	Lipid peroxidation
MS	Multiple sclerosis
MW	Molecular weight
NSAID	Non-steroidal anti-inflammatory agent
ROS	Reactive Oxygen Species
TAC	Total antioxidant capacity
TC	Total cholesterol
TG	Triglycerides
TPSA	Topological polar surface area
