# Supplementary material for: From Lipid Regulation to Neuroprotection: Multitarget (Benzo)thiazine Derivatives as Promising Leads
Source: Molecules. 2025 Nov 25;30(23):4542. doi: 10.3390/molecules30234542 (PMC12693416; doi:10.3390/molecules30234542)
Supplement: Supplementary file 1 [file molecules-30-04542-s001.zip › molecules-3970230-supplementary.pdf]

molecules

MDPI

Supplementary material

Table S1: Structures of the new compounds

Compound	IUPAC name	Structure
**1**	(*E*)-1-(2-([1,1′-biphenyl]-4-yl)thiomorpholino)-3-(4-hydroxy-3-methoxyphenyl)prop-2-en-1-one	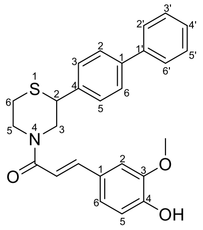
**2**	(*E*)-1-(2-([1,1′-biphenyl]-4-yl)thiomorpholino)-3-(3,4-dimethoxyphenyl)prop-2-en-1-one	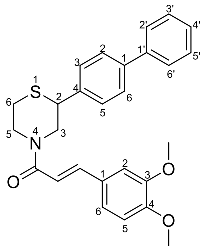
**3**	(2-([1,1′-biphenyl]-4-yl)thiomorpholino)(3,4,5-trihydroxyphenyl)methanone	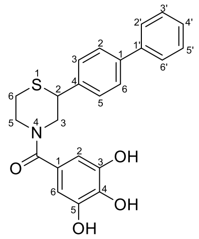
**4**	(2-([1.1′-biphenyl-4-yl)thiomorpholino)(3,4,5-trimethoxyphenyl)methanone	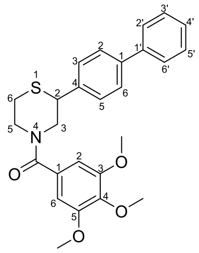
**5**	(2-([1,1′-biphenyl]-4-yl)thiomorpholino)(3,5-ditertbutyl-4-hydroxyphenyl)methanone	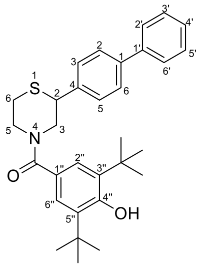

Compound	IUPAC name	Structure
**6**	(*E*)-1-(2-([1.1′-biphenyl]-4-yl)thiomorpholino)-3-(3,5-ditertbutyl-4-hydroxyphenyl)prop-2-en-1-one	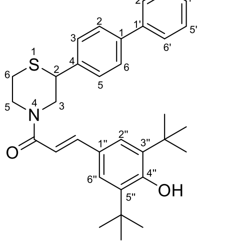
**7**	1-(2-([1,1′-biphenyl]-4-yl)thiomorpholino)-2-(4-isobutylphenyl)propan-1-one	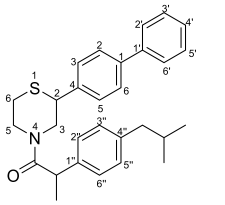
**8**	(*E*)-1-(2-([1,1′-biphenyl]-4-yl)thiomorpholino)-3-phenylprop-2-en-1-one	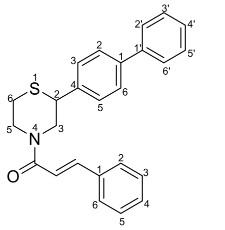
**9**	(2-([1,1′-biphenyl-4-yl]thiomorpholino)(pyridine-3-yl)methanone	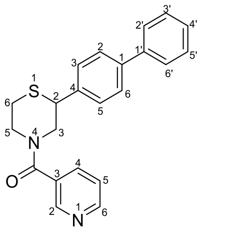
**10**	(2-([1,1′-biphenyl-4-yl]thiomorpholino)(6-hydroxy-2,5,7,8-tetramethylchroman-2-yl)methanone	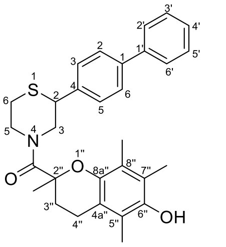
**11**	2-([1,1′-biphenyl]-4-yl)-3,4-dihydro-2H-benzo[b][1,4]thiazine	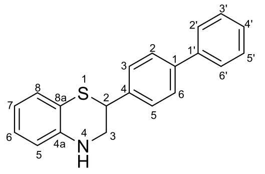
**12**	(*E*)-1-(2-([1,1′-biphenyl]-4-yl)-2,3-dihydro-4H-benzo[b][1,4]thiazin-4-yl)-3-(3,4-dimethoxyphenyl)prop-2-en-1-one	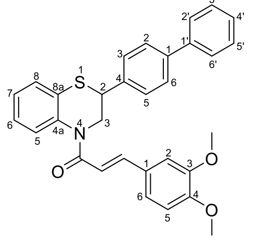

Compound	IUPAC name	Structure
**13**	(2-([1,1′-biphenyl]-4-yl)-2,3-dihydro-4H-benzo[b][1,4]thiazin-4-yl)(3,4,5-trihydroxyphenyl)methanone	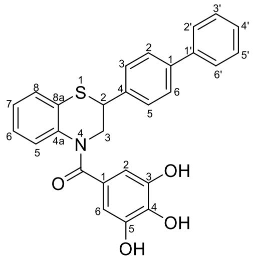
**14**	(2-([1,1′-biphenyl]-4-yl)-2,3-dihydro-4H-benzo[b][1,4]thiazin-4-yl)(3,4,5-trimethoxyphenyl)methanone	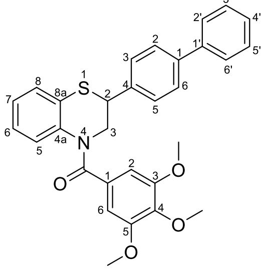
**15**	(2-([1,1′-biphenyl]-4-yl)-2,3-dihydro-4H-benzo[b][1,4]thiazin-4-yl)(3,5-ditertbutyl-4-hydroxyphenyl)methanone	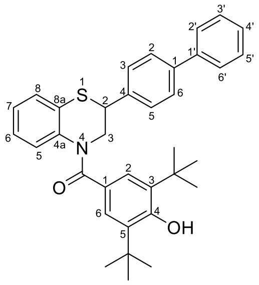
**16**	(*E*)-1-(2-([1,1′-biphenyl]-4-yl)-2,3-dihydro-4H-benzo[b][1,4]thiazin-4-yl)-3-(3,5-ditertbutyl-4-hydroxyphenyl)prop-2-en-1-one	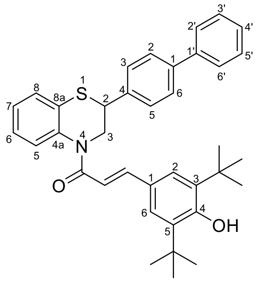
**17**	1-(2-([1,1′-biphenyl]-4-yl)-2,3-dihydro-4H-benzo[b][1,4]thiazin-4-yl)-2-(4-isobutylphenyl)propan-1-one	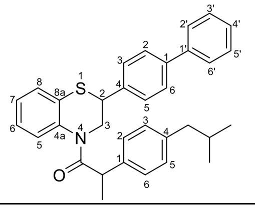
